# Hybrid-Driven Bacillus Calmette–Guérin Carrier for Targeted Immuno-Chemo Combo Therapy in Bladder Cancer

**DOI:** 10.34133/cbsystems.0492

**Published:** 2026-04-28

**Authors:** Zhanxiang Zhang, Lin Wang, Zhongcheng An, Yuhang Jiang, Jiawen Niu, Laishou Yang, Qianqian Wang, Xinjian Fan, Tianlong Li

**Affiliations:** ^1^ State Key Laboratory of Robotics and System, Harbin Institute of Technology, Harbin 150000, China.; ^2^ Suzhou Research Institute of HIT, Suzhou 215104, China.; ^3^ Jiangsu Key Laboratory for Design and Manufacturing of Precision Medicine Equipment, School of Mechanical Engineering, Southeast University, Nanjing 211189, China.; ^4^ School of Mechanical and Electrical Engineering, Soochow University, Suzhou 215137, China.

## Abstract

Combination therapy is a promising approach to enhancing antitumor efficacy and overcoming multidrug resistance. Intravesical instillation of Bacillus Calmette–Guérin (BCG) combined with chemotherapy has been employed to improve bladder cancer treatment efficacy, but outcomes are often limited by high-dose drug irritation, poor patient tolerance, and insufficient targeting. To overcome these limitations, we propose a microrobot (MR)-based targeted drug delivery strategy for precise co-delivery of BCG and paclitaxel to bladder tumors, facilitating sustained drug release and minimizing off-target effects. The MRs are fabricated using a layer-by-layer assembly technique, incorporating antitumor drugs, magnetic nanoparticles, and viable BCG. Under the synergistic action of external magnetic fields and hydrogen microbubbles generated through chemical reactions, the MRs achieve targeted navigation and effective accumulation within the 3-dimensional tumor microenvironment. Subsequently, the combined chemotherapeutic and immunostimulatory effects effectively inhibit tumor progression. This approach not only minimizes off-target effects but also facilitates sustained drug release. Additionally, a wearable magnetic fixation device based on a Halbach array is employed to fixate the MRs at the targeted region, further improving drug retention and enhancing therapeutic efficacy. The experimental results demonstrate that this MR-based delivery system holds considerable potential for clinical translation into combination therapies for bladder cancer.

## Introduction

Bladder cancer ranks as the 4th most common malignancy among males and the 11th among females worldwide, with annually increasing incidence rates, imposing substantial global health and economic burdens [1,2]. Transurethral resection of bladder tumors remains the standard clinical approach for bladder cancer management. However, patients face high recurrence rates, exceeding 75% within 5 years posttreatment [3,4], necessitating the development of intravesical drug delivery or immunomodulatory strategies to effectively reduce recurrence and prevent metastasis. Traditional therapeutic infusion methods suffer from inadequate targeting and considerable systemic side effects, negatively affecting patient health [4]. Moreover, intravesically injected therapeutics can be rapidly cleared from the bladder due to frequent urination, necessitating repeated administrations that may cause infections and inflammation. Long-term use of monotherapy further raises concerns regarding drug resistance and therapeutic efficacy, particularly considering inter-individual variability in drug responses [5]. Recently, several intravesical delivery regimens and platforms combining Bacillus Calmette–Guérin (BCG) with chemotherapeutic agents have been developed, demonstrating enhanced local antitumor activity and reduced systemic toxicity. However, these combination strategies are still constrained by suboptimal lesion targeting and insufficient bladder residence time, as urinary washout and limited tissue adhesion compromise the durability of therapeutic response. Thus, improving drug targeting and prolonging intravesical residence remain key priorities for further optimization and clinical application [6–9].

Micro/nanorobots, engineered at the micro- and nanoscale, exhibit great potential for targeted drug delivery [10–13] due to their controllable locomotion capabilities under external physical fields (such as optical [14–17], acoustic [18], and magnetic [19–21]) or chemical-reaction-induced propulsion [22–24]. They demonstrate substantial advantages in intelligent navigation [25–30], barrier penetration [31–33], and efficient drug delivery [34–37], thus emerging as promising platforms for synergistic cancer treatment [38–40]. Recently, multifunctional microrobot (MR)-based drug delivery systems have been extensively developed [41–43], integrating chemotherapy drugs and immunomodulators to achieve synergistic effects [44], effectively improving anticancer outcomes, and markedly mitigating drug resistance [6,45–47]. Furthermore, this combination therapy approach reduces the variability of drug responses among individuals, enhancing the overall clinical applicability of therapeutic regimens. However, micro/nanorobots still face substantial challenges in achieving efficient drug delivery within complex 3-dimensional biological environments. Chemical propulsion suffers from random movement, hindering precise drug delivery, while magnetic-field-driven systems, despite offering accurate navigation and excellent biocompatibility, generally require strong magnetic fields or a high magnetic material content, leading to manufacturing complexity and limited practical applications. Therefore, effectively combining magnetic and chemical propulsion methods while concurrently overcoming the instability and randomness associated with chemically generated bubbles [48–50], to achieve stable and reliable 3-dimensional movement control, remains an urgent issue [51,52]. Moreover, due to the frequent urination inherent to bladder physiology, it is challenging to ensure long-term stable retention of MRs and drugs within the bladder through surface modification alone. Consequently, developing an MR system that integrates precise directional control, effective tumor targeting, stable retention, and multifunctional therapeutic capabilities is a critical yet unmet need in the treatment of bladder cancer.

**Fig. 1.**
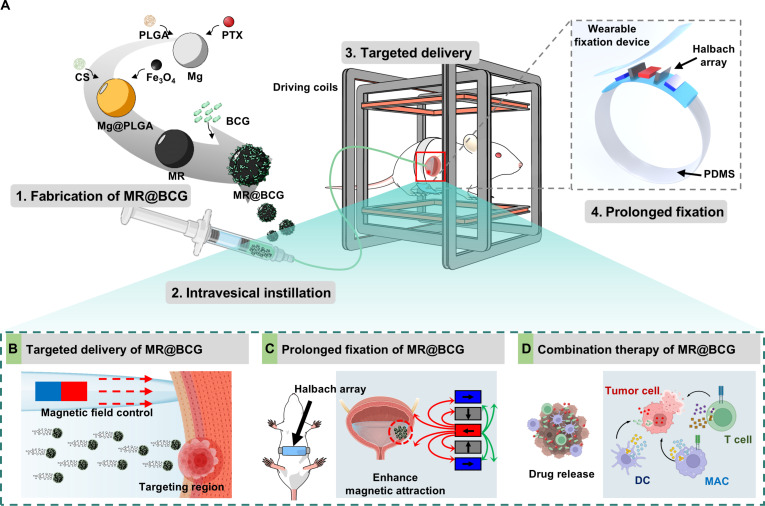
Schematic illustration of intravesical treatment using a microrobot loaded with Bacillus Calmette–Guérin (MR@BCG). (A) Schematic illustration of MR@BCG fabrication and the magnetic actuation/retention setup for intravesical therapy. (B) Magnetically driven targeted navigation of MR@BCG in the bladder toward the tumor region. (C) Prolonged retention of MR@BCG at the lesion site enabled by a Halbach magnetic array. (D) BCG-mediated immune activation by MR@BCG to enhance antitumor responses. CS, chitosan; MR, microrobot; DC, dendritic cell; MAC, macrophage.

This study introduced a hybrid-propulsion system combining magnetic fields and chemical reactions to navigate MRs for the targeted delivery of the anticancer drug paclitaxel (PTX) and the immunomodulator BCG, enabling combined tumor therapy (as shown in Fig. 1). Specifically, MRs were fabricated using magnesium (Mg) microparticles as the core, coated with poly(lactic-*co*-glycolic acid) (PLGA) and chitosan (CS) layers. Meanwhile, PTX and Fe_3_O_4_ were respectively loaded onto the PLGA and CS layers. Subsequently, BCG was loaded onto the surface of the MR through electrostatic interactions. After urethral infusion into the bladder, the MRs exhibited a hybrid-propulsion mechanism. Bubbles generated through the reaction of Mg with water provided a powerful thrust, enabling robust 3-dimensional motion and allowing the MRs to navigate against gravity and reach arbitrary locations within the bladder. Simultaneously, the torque generated by the magnetic field ensured precise directional control, while the magnetic gradient force further assisted propulsion, enhancing the MRs’ ability to efficiently traverse complex bladder environments and accurately reach tumor sites. Upon reaching the tumor, the CS layer on the MR surface exhibited excellent mucoadhesive properties, facilitating adhesion to the bladder wall. To further improve fixation stability at the tumor site, a Halbach array was employed to generate an enhanced gradient magnetic field, ensuring effective MR immobilization in the targeted region. This approach markedly prolonged MR residence time in the bladder, optimizing drug delivery efficiency. Subsequently, BCG facilitated the recruitment and activation of immune cells, effectively suppressing tumor growth. Furthermore, we evaluated the in vivo therapeutic efficacy of MRs loaded with BCG (MR@BCG) using an orthotopic bladder cancer model. The results demonstrated that these MR@BCGs markedly enhanced drug bioavailability and action duration. The co-delivery of PTX and BCG via an MR platform enabled effective chemotherapeutic and immunotherapeutic synergy for potent antitumor efficacy, positively impacting the continued development of clinical disease management strategies.

## Materials and Methods

### MB49

MB49 cells were purchased from Procell. The cells were cultured at 37 °C in a humidified atmosphere containing 5% CO_2_ in cell culture dishes using Dulbecco’s modified Eagle medium (DMEM; Procell) supplemented with 10% (v/v) fetal bovine serum (HyClone) and 1% (v/v) penicillin–streptomycin.

### Fabrication of MR@BCG

First, a 2% polyvinylpyrrolidone solution was uniformly applied onto a glass slide and subsequently dried at 60 °C for 10 min. Mg microspheres were uniformly dispersed onto the glass slide, exposed to air at 80% relative humidity at 25 °C for 5 s, followed by drying at 90 °C for 30 min. Loose particles that were not fixed were removed via gentle blowing to ensure partial embedding of the Mg particles in the polyvinylpyrrolidone film. Next, an ethyl acetate solution containing 2% PLGA and different PTX concentrations was applied to the glass slide. After drying, a deionized water solution containing 3% Fe_3_O_4_, 0.05% CS, 0.1% sodium dodecyl sulfate, and 0.02% acetic acid was prepared. Then, the mixture was coated onto the glass slide. After drying and directional magnetization, the MRs were collected into a centrifuge tube. Subsequently, BCG was added to the MRs at a 1:4 mass ratio and stirred in a deionized water solution for 30 min. The MR@BCG samples were washed twice with deionized water and dried. The final MR@BCG samples were stored at −20 °C for further use.

### Characterization of MR@BCG

Scanning electron microscopy (SEM) images of Mg@PLGA, MR, and MR@BCG were acquired using a scanning electron microscope (Thermo Fisher Scientific). Energy-dispersive x-ray (EDX) spectral analysis was performed using an Oxford EDS detector (Xplore 30) coupled to SEM. To confirm successful BCG loading, fluorescent MR@BCG was prepared following the same protocol, where the PLGA layer was labeled with 1,1′-dioctadecyl-3,3,3′,3′-tetramethylindotricarbocyanine iodide (DiR) and BCG with fluorescein isothiocyanate. Fluorescence images were captured using an Olympus microscope. The particle size distribution of the MRs was statistically analyzed by measuring 200 individual particles under an optical microscope, and the hysteresis loops of MRs were measured using a vibrating-sample magnetometer (Lake Shore). The zeta potentials of Mg, Mg@PLGA, MR, and MR@BCG were measured using a zeta potential analyzer (Malvern). To determine the loading efficiency of BCG on MRs, the MRs were resuspended in a 2% sodium dodecyl sulfate solution and briefly vortexed. Subsequently, the suspension was incubated at 37 °C for 30 min and subjected to 2 min of sonication. The sample was centrifuged at 3,000 rpm for 5 min, and the protein content of the supernatant was analyzed using a bicinchoninic acid protein assay kit (Beyotime). The background protein content of an equivalent quantity of MRs without BCG loading was subtracted from each measurement to calculate the final loading efficiency. BCG-loaded MRs were incubated in urine at room temperature for 20 min under gentle agitation. MRs were then centrifuged at 3,000 rpm for 5 min, washed once with phosphate-buffered saline (PBS), and the retained BCG was quantified by bicinchoninic acid using the same protocol as the loading efficiency assay. Retention was calculated relative to the initially loaded amount.

### Encapsulation efficiency and release profile of PTX in the MR

The encapsulation efficiency and drug release profile of PTX were evaluated as follows: The PTX standard curve was established using a high-performance liquid chromatography (HPLC) system fitted with a C18 chromatographic column. The mobile phase was composed of methanol, water, and acetonitrile (23:41:36 ratio), at a flow rate of 1 ml/min. Detection was performed at 227 nm, and the column temperature was set at 35 °C. To assess the encapsulation efficiency, the MRs were immersed in 2 ml of methanol, ultrasonically disrupted for 10 min, and shaken overnight to ensure complete PTX release. The concentration of PTX in the solution was quantified using the HPLC system. For the drug release profile, the MRs were incubated in PBS and urine at 37 °C to facilitate gradual PTX release. At predetermined time points, 2 ml of the solution was collected, centrifuged, and analyzed for PTX concentration using HPLC. Finally, the encapsulation efficiency and drug release profile of PTX were calculated and plotted based on the standard curve.

### Tumor in vitro killing effect of MRs

MB49 cells were seeded into a 96-well plate at a density of 3 × 10^3^ cells per well and incubated overnight. Subsequently, bare MRs, free PTX, and PTX-loaded MRs were added to the wells and incubated for 4, 24, and 48 h. The cell viability at each time point was measured using the Cell Counting Kit-8 (CCK-8) assay (Beyotime). The absorbance at 490 nm was measured with a microplate reader. Similarly, a cell death assay was conducted using propidium iodide (PI; Beyotime). Cells were incubated with bare MRs, free PTX, and PTX-loaded MRs for 24 h. After the incubation, the supernatant was removed and the cells were stained with a PI solution for 30 min. After 2 washes with PBS, the cells were observed under a fluorescence microscope.

### PTX effects on immune/tumor cells

In this study, immune cells were isolated from the spleens of C57BL/6 mice (6 to 8 weeks, specific-pathogen-free housing; protocols approved by the institutional animal care and use committee). Briefly, spleens were aseptically harvested into ice-cold PBS and mechanically dissociated through a 70-μm cell strainer to obtain single-cell suspensions. Red blood cells were lysed with ammonium–chloride–potassium buffer at room temperature for 2 to 3 min, the reaction was quenched with complete medium, and cells were washed by centrifugation (300 to 400 g, 5 min). Cultured MB49 bladder cancer cells (prepared as described above) and splenic immune cells were seeded into 96-well plates and treated for 48 h with graded concentrations of PTX prepared from a dimethyl sulfoxide stock. Cell viability was then assessed using the CCK-8 assay by adding 10 μl of reagent per well and incubating at 37 °C for 1 to 2 h, followed by measurement of optical density at 450 nm on a microplate reader.

### Immune cell activation

T cells and macrophages were isolated from the spleen and bone marrow of C57BL/6 mice. These cells were cultured with bare MRs, BCG, and MR@BCG for 24 h at 37 °C in a humidified 5% CO_2_ atmosphere. The activation of T cells and macrophages was evaluated by flow cytometry using antibodies against CD4^+^, CD8^+^ T cells, CD86^+^, and CD206^+^ macrophages. Additionally, enzyme-linked immunosorbent assays were performed to quantify tumor necrosis factor-α (TNF-α), interferon-γ (IFN-γ), interleukin-2 (IL-2), and interleukin-6 (IL-6) levels in the supernatants.

### Intelligent navigation system of MR@BCG

The intelligent navigation system of MR@BCG was composed of a decoupled direction and speed controller, a tracker, and a magnetic field generator. The motion trajectories of the MRs were acquired at 50 frames per second using an IX73 inverted microscope (Olympus, Tokyo, Japan) paired with a Point Gray charge-coupled device camera (GS3-U3-51S5C/M-C, FLIR, Wilsonville, OH, United States). The trajectories were tracked using a YOLOv5-based object detection algorithm integrated with the DeepSORT tracking framework. The position and velocity of the MRs were determined based on the tracking results. A velocity controller and a direction controller were employed to generate control signals for correcting velocity and direction errors. After receiving the control signals, the data acquisition board (DAQ, NI-PCI-6259) generated the driving signals, which were amplified by power amplifiers and transmitted to the electromagnetic coils. The electromagnetic coils consisted of triaxial Helmholtz coils and triaxial Maxwell coils to generate desired rotating and gradient magnetic fields.

### Controllable locomotion of MR@BCG in microchannels and bladders

The experiment was conducted using a polydimethylsiloxane-based microfluidic device. DiR-labeled MRs were added to the left chamber and treated without or with magnetic field control for 5 min. Fluorescent images of the MR distribution in the microfluidic channel were captured using an in vivo fluorescence imaging system (Tanon). Similarly, the urinary system of C57BL/6 mice was dissected and formalin-fixed, and the C57BL/6 mice were anesthetized. The MRs were resuspended in PBS solution and injected into the ex vivo and in vivo bladder of C57BL/6 mice from the urethra via the urethral catheter. The MRs were processed without or with magnetic field control for 5 min. Fluorescent images of the MR distribution in the ex vivo and in vivo bladder were captured using an in vivo fluorescence imaging system.

### The fixation of MRs

After dissecting the urinary system of mice, the tissue was formalin-fixed. DiR-labeled MRs and DiR–PLGA particles were injected into the bladder via the urethra using an intravenous catheter. The bladder was gently compressed to mimic urination. After each urination cycle, the fluorescent images of the MR distribution in the bladder were captured using an in vivo fluorescence imaging system, and the retention ratio was calculated. Similarly, after anesthetizing the mice, DiR-labeled MRs and DiR dye were injected into the bladder using an intravenous catheter. The distribution of the MRs in the bladder was regularly monitored through fluorescence imaging, both with and without the use of the retention device. The retention ratio was calculated based on the fluorescent images.

### In vivo antitumor study of MR@BCG

After anesthetizing C57BL/6 mice, 40 μl of PBS containing 10^6^ luciferase (LUC)-labeled MB49 bladder cancer cells were injected into the bladder wall to establish the orthotopic bladder tumor model. Seven days after the cell inoculation, the mice were divided into 8 groups and injected with PBS, bare MRs, PTX, BCG, MR@BCG, MR@PTX (without BCG) + MF, MR@BCG (without PTX) + MF, and MR@BCG + MF. The intravesical administration lasted for 1 h, during which the mice remained under anesthesia. For the MR@BCG + MF group, after intravesical instillation of MR@BCG, a magnetic field was applied to drive the MRs toward the tumor site, and the mice wore a retention device. Such intravesical administrations were performed every 3 d. The orthotopic tumor growth was monitored every 2 d using bioluminescence imaging with an in vivo fluorescence imaging system. After finishing the treatment, the mice were euthanized, and the bladders were excised for histological analysis. Hematoxylin and eosin staining, Ki-67 immunostaining, and immunofluorescence staining of CD8^+^ T cells and CD86^+^ macrophages were performed to observe tumor growth features and immune cell activation.

### Ethics statement

All animal procedures were reviewed and approved by the Animal Welfare and Ethics Committee of the Harbin Institute of Technology (Approval No. 25YS-092, dated 2025 March 4) and were conducted in accordance with institutional guidelines and the Regulations for the Administration of Affairs Concerning Experimental Animals (China).

## Results

### Fabrication and characterization of MR@BCG

**Fig. 2.**
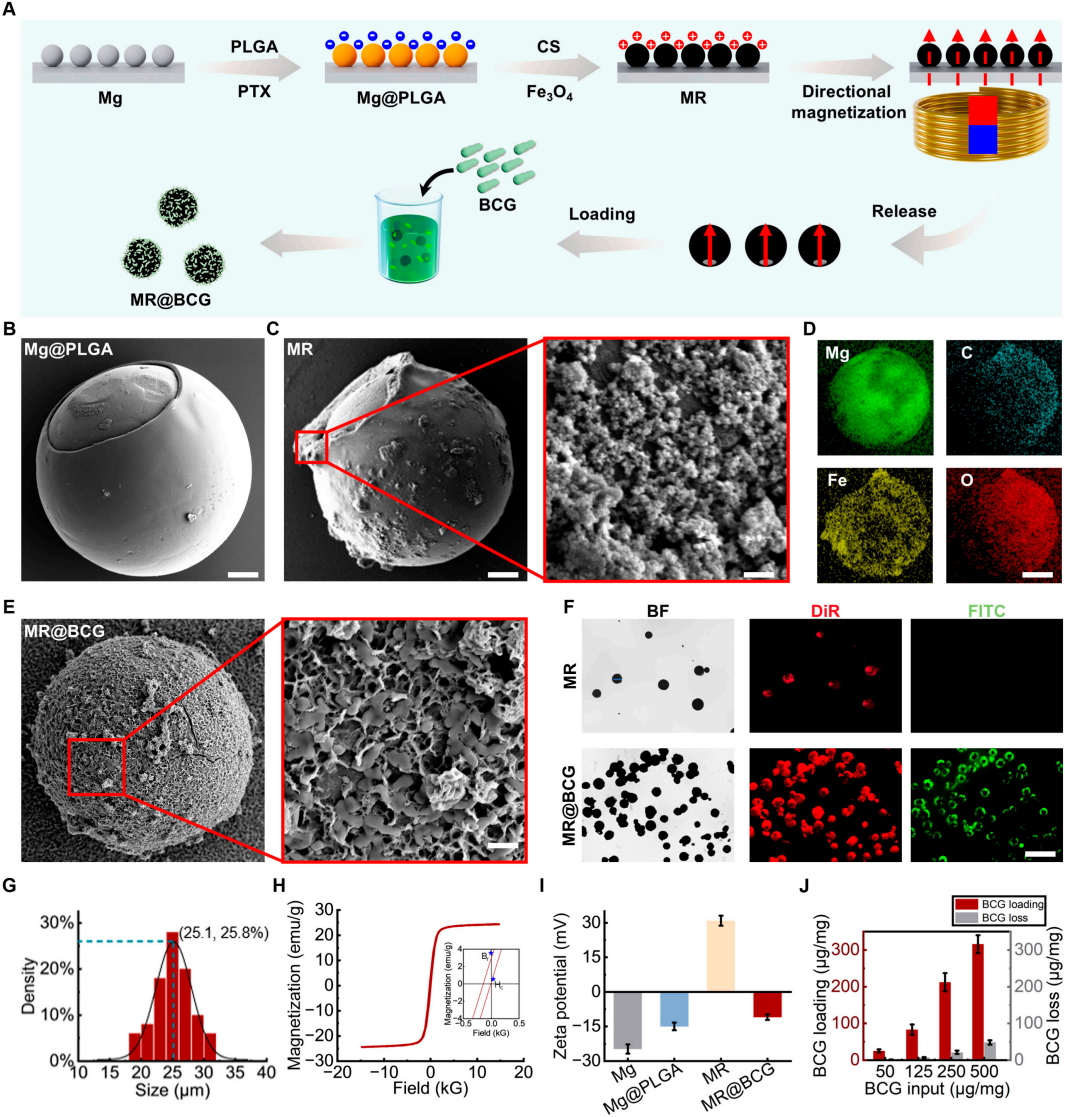
Fabrication and characterization of MR@BCG. (A) Schematic illustration of the MR@BCG fabrication process. (B to D) The Scanning electron microscopy (SEM) and energy-dispersive x-ray (EDX) images of MRs. Scale bars: (B) 3.5 μm, (C) 3.5 μm and 200 nm, and (D) 7.5 μm. (E and F) SEM, optical, and fluorescence images of MR@BCG. Scale bars: (E) 3.5 and (F) 70 μm. (G) Particle size distribution of MRs. (H) Vibrating-sample magnetometer (VSM) curve of MR@BCG showing magnetic properties. (I) The zeta potentials of Mg, Mg@PLGA, MR, and MR@BCG. (J) Quantification of BCG on MR@BCG (per 1 mg microrobots) across varying initial BCG inputs, including the initially loaded amount and the amount retained after 20-min incubation in urine. FITC, fluorescein isothiocyanate; BF, bright-field; DiR, 1,1′-dioctadecyl-3,3,3′,3′-tetramethylindotricarbocyanine iodide.

The MRs were fabricated through a layer-by-layer self-assembly process (Fig. 2A). First, Mg microparticles (~25 μm in diameter) were uniformly dispersed onto a glass slide. A PLGA layer containing PTX was then coated onto the Mg particles, covering most of their surface area while leaving small openings at the contact points with the glass slide. These exposed Mg sites reacted with the surrounding aqueous environment, generating the hydrogen gas required for the MR’s propulsion. Simultaneously, the antitumor drug PTX was efficiently encapsulated. A CS layer containing Fe_3_O_4_ nanoparticles was subsequently added on top of the PLGA layer to facilitate MR manipulation in response to an external magnetic field and create a positively charged surface. After the CS layer was assembled, the MRs were magnetized along an axis opposite to the openings, allowing for precise directional control using the external magnetic field. Then, BCG was successfully loaded through electrostatic interactions. Finally, the free MR@BCGs were washed out from the solution, vacuum-dried, and stored at −20 °C for subsequent use.

SEM images were employed to visualize the surface morphology of the MRs at each stage of the preparation process. As shown in Fig. 2B, a uniform PLGA layer containing the drug was successfully coated onto the surface of the Mg particles. A distinct small aperture was observed in the PLGA layer, corresponding to the contact point between the Mg particles and the glass substrate during fabrication. This small aperture allowed Mg to react with water from the external environment, producing hydrogen gas, which was expelled through the opening, resulting in unidirectional thrust and driving effective MR motion. Furthermore, Fig. 2C presents SEM images of the MRs, confirming the successful encapsulation of the CS layer containing iron(III) oxide (Fe_3_O_4_). EDX spectroscopy and chemical mapping were performed to confirm the presence of magnesium, Fe_3_O_4_, and organic layers, verifying their successful incorporation (Fig. 2D). The Fe_3_O_4_ component enabled the MRs to respond to external magnetic fields, facilitating motion control. Similarly, the SEM and EDX images revealed the presence of a small opening, confirming the successful construction of a Janus structure. The CS coating imparted a positive charge to the MR’s surface, enabling electrostatic interaction with the negatively charged BCG vaccine, thereby achieving efficient loading, as shown in Fig. 2E. Additionally, both the CS and BCG layers were fluorescently labeled to confirm their presence within the MRs (Fig. 2F). CS was tagged with DiR, and BCG was labeled with fluorescein isothiocyanate. Fluorescence microscopy observations revealed red fluorescence on the MRs, confirming successful CS coating. After incubating the MRs with BCG, green fluorescence was observed, indicating the successful loading of BCG onto the carriers. Beyond serving as a carrier for the lipophilic drug PTX, the CS layer also enabled efficient loading of the water-soluble anticancer drug doxorubicin (DOX). As shown in Fig. S1, the characteristic red fluorescence of DOX was co-localized with the MRs, confirming successful DOX loading.

The average diameter of the MRs was determined by measuring 200 individual particles, resulting in 25.1 μm, as shown in Fig. 2G. The coating of PLGA and CS layers showed no noticeable impact on the particle size. The MRs were magnetized along their long axes (perpendicular to the small opening) using a magnetizer, and their magnetic properties were subsequently characterized using a vibrating-sample magnetometer. As illustrated in Fig. 2H, the magnetization curves exhibited ferromagnetic-like characteristics, including notable remanence and coercivity, indicating stable magnetic moments. Such hysteresis likely arose from interparticle interactions among immobilized ∼20 nm Fe_3_O_4_ nanoparticles (dipolar/occasional exchange coupling) within the polymer matrix, which effectively blocked the superspins at room temperature [53]. Due to these stable magnetic moments, the MRs demonstrated reliable directional locomotion under an external magnetic field. Additionally, to confirm successful BCG loading, the zeta potential of the MRs was measured at each stage of the fabrication process (Fig. 2I). After PLGA coating, the MRs exhibited a distinct negative surface charge, whereas subsequent CS coating conferred a positive charge, thereby facilitating electrostatic adsorption of negatively charged BCG. Following successful loading, the MRs displayed a negative surface charge attributed to the immobilized BCG. Furthermore, to optimize BCG loading efficiency, different BCG-to-MR ratios were tested (Fig. 2J), with error bars representing the standard deviation from 5 independent experiments; 250 μg of BCG per 1 mg of MRs was identified as the optimal ratio for achieving the best balance between loading yield and efficiency. On the other hand, we experimentally evaluated the stability of BCG association with the MRs in urine. After 20 min, approximately 85% of the initially loaded BCG remained adsorbed on the MRs, indicating stable adsorption in urine.

### Intelligent navigation of MRs via hybrid-driven strategies

**Fig. 3.**
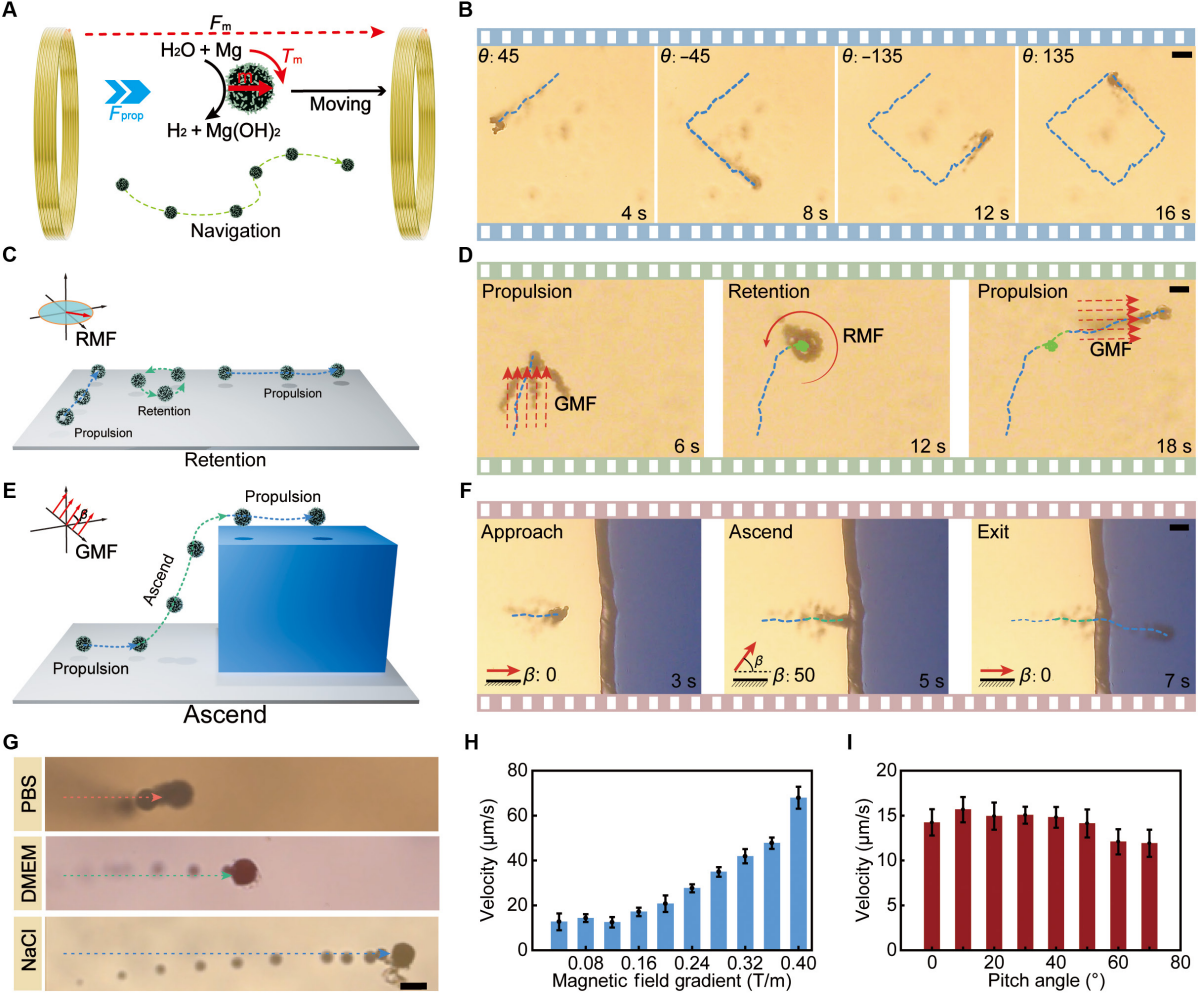
Navigation of the MR. (A) Schematic illustration of MR control principles. (B) Time-lapse imaging of MR autonomous navigation under system control. Scale bar: 30 μm. (C) Schematic illustration of the retention locomotion of MRs under a rotating magnetic field. (D) Time-lapse images showing MR retention under a rotating magnetic field. Scale bar: 40 μm. (E) Schematic illustration of the obstacle-crossing locomotion of MRs actuated by gradient magnetic fields with a pitch angle. (F) Time-lapse snapshots of 3-dimensional (3D) navigation. Scale bar: 30 μm. (G) Time-lapse images of MRs moving in different solutions. Scale bar: 25 μm. (H and I) Relationship between MR velocity and magnetic field gradient and pitch angle. RMF, rotating magnetic field; GMF, gradient magnetic field; DMEM, Dulbecco’s modified Eagle medium; PBS, phosphate-buffered saline.

The previously developed Mg-based MR drug delivery system, although capable of generating propulsion through hydrogen bubbles, lacked precise motion control. Due to the inherent randomness in its movement, the Mg-based MR exhibited limited diffusion distance and struggled to accumulate in targeted regions. To address this, Fe_3_O_4_ nanoparticles were incorporated into the CS coating of the MR and then magnetized along its long axis (perpendicular to the small opening), thereby enabling precise directional control under the external magnetic field. Specifically, during operation, a gradient magnetic field was applied, generating torque that aligned the MR’s long axis with the direction of the magnetic field, thus producing directional movement. Additionally, the gradient magnetic field exerted a propulsive force to facilitate the movement of the MRs (as shown in Fig. 3A). Inertial effects were neglected due to the low-Reynolds-number environment, and the dynamic model was described as follows [20]:

$$\left\{\begin{array}{c} F_{\text{prop}}\;\text{cos}\left(\theta +\delta \right)+F_{m_{x}}=F_{{\text{viscous}}_{x}}\\ F_{\text{prop}}\;\text{sin}\left(\theta +\delta \right)+F_{m_{y}}=F_{{\text{viscous}}_{y}}\\ T_{\text{prop}}+T_{m}=T_{\text{viscous}} \end{array}\right.$$ (1)

where *F*_prop_ represents the magnitude of the jet propulsion force, *θ* denotes the current orientation of the MR, and *δ* indicates the propulsion deflection angle. Additionally, $F_{m_{x}}$ and $F_{m_{y}}$ refer to magnetic forces acting along the *x*- and *y*-directions, respectively. Translational viscous drag forces are represented by $F_{{\text{viscous}}_{x}}$ in the *x*-direction and $F_{{\text{viscous}}_{y}}$ in the *y*-direction. Furthermore, the considered torques included the propulsion-induced torque *T*_prop_, the magnetic torque *T*_m_, and the rotational viscous drag torque *T*_viscous_.

Magnetic force and torque could be calculated using the magnetic field (*B*) and magnetic field gradient (∇*B*) as follows [20,52]:

$$F_{m}=V\left(M\cdot \nabla \right)B$$ (2)

$$T_{m}=VM\times B$$ (3)

where *V* is the volume of the magnetized object and *M* is the uniform magnetization of the magnetic material.

However, challenges persisted with unpredictable temporal variations in the magnitude and direction of the jet propulsion force, as well as random shifts in the robot’s centroid due to the gradual consumption of the magnesium core. Consequently, as a nonlinear and time-varying system, controlling the motion of the MR remained a complex task. To address these challenges, we designed a control system based on an extended state observer to estimate disturbances and employ magnetic field to compensate for uncertainties in bubble-driven propulsion, thereby achieving precise 3-dimensional locomotion, as detailed in Texts S1 and S2 and illustrated in Figs. S2 and S3.

Compared to the uncontrolled random movement, the diffusion distance of the MRs with directional control increased approximately 4-fold, as shown in Fig. S4. Furthermore, in vitro time-lapse microscopic images (as shown in Fig. 3B) presented the trajectory of the MR’s self-correction under system control. During motion, digital microscopy data for visual localization of the MR were fed into the control system to detect directional errors. Furthermore, the controller processed this information and issued commands to adjust the robot’s movement, ensuring adherence to the predetermined trajectory. Through continuous feedback control, precise navigation of the MR was successfully achieved. Fig. S5 illustrates the direction deviation observed throughout the process. As shown in Fig. S6, the average directional error measured was 6.76°.

Chemically actuated MRs commonly encounter challenges in achieving stable and precise retention due to the inherent unpredictability of chemical reactions. To overcome this limitation, we propose a magnetically controlled retention strategy. Specifically, active retention of MRs was achieved using rotating magnetic fields, as illustrated in Fig. 3C and D. Initially, the MR was guided toward the designated region under a gradient magnetic field. Upon reaching the targeted position, a rotating magnetic field was applied in the *x*–*y* plane, causing the MR to rotate and hover stably in place. During each rotation cycle, the propulsion forces generated by bubbles canceled each other out, resulting in negligible net displacement. Importantly, this hovering mechanism effectively prevented the MR from drifting away from the targeted location, thus substantially extending its retention time. When the rotating magnetic field was deactivated and a horizontal gradient was reapplied, the MR transitioned smoothly from retention to locomotion, resuming motion in the specified direction.

Moreover, the integration of bubble-generated propulsion with a gradient magnetic field not only counteracts gravitational forces but also enables precise 3-dimensional navigation of the MR. As shown in Fig. 3E and F, when a horizontal gradient magnetic field was applied to the right, the MR moved toward the obstacle along the bottom surface. By tilting the magnetic field at 50° relative to the *x*–*y* plane, the MR underwent rotation in response to the magnetic torque. The propulsion force generated by the bubble, in combination with the gradient magnetic force, counteracted gravity, enabling the MR to move upward along the magnetic field direction and ultimately surmount the obstacle measuring 100 μm in height. This 3-dimensional mobility enabled the MR to deliver drugs within various complex biological tissues and organs, notably expanding its potential for biomedical applications [51,54,55].

Next, we further explored the propulsion performance of MRs in various biological fluids, as shown in Fig. 3G. The MRs demonstrated effective movement in PBS, physiological saline, and DMEM. Under the same magnetic field conditions, MRs achieved the highest velocity of 32 μm/s in physiological saline, and the lowest in PBS, at 12 μm/s. This was likely due to organic molecules and proteins adsorbing onto the Mg surface and partially obstructing Cl^−^ adsorption, whereas the phosphate ions in PBS rapidly precipitate as an insoluble Mg_3_(PO_4_)_2_ layer that shields the metal from chloride attack and markedly slowed hydrogen-generating reaction. Furthermore, we evaluated the influence of environmental pH on MR propulsion under bladder-relevant conditions. To encompass clinically encountered urine milieus—acidic (pH 4.5 to 6), near-neutral (pH 7), and alkaline (pH 8, urease-positive infection)—we tested pH values of 4.5, 6.0, 7.0, and 8.0. The corresponding mean velocities were 24.56, 17.48, 12.76, and 9.81 μm/s, respectively (Fig. S7), indicating enhanced propulsion in acidic media and diminished propulsion in alkaline media. This behavior aligns with the dissolution or stabilization of the Mg(OH)_2_ passivation layer, which respectively promotes or suppresses hydrogen bubble propulsion. Additionally, we statistically evaluated the reaction duration of MRs in different solutions. In physiological saline, the reaction was the fastest, lasting approximately 2 min, while in DMEM and PBS, the reaction lasted about 3 min. These reaction durations were sufficient for targeted drug delivery within a mouse bladder, considering the average mouse bladder diameter of approximately 4 to 6 mm. Moreover, propulsion velocity and delivery distance could be further improved by increasing the Mg sphere diameter or by applying gradient magnetic fields for additional propulsion. Notably, once the Mg core was exhausted, gradient fields alone were sufficient to continue propelling the MRs (Movie S4), thereby fulfilling the motility and distance requirements for large-animal or clinical scenarios. To determine optimal conditions for such magnetic propulsion and fully leverage the potential of gradient magnetic fields, it is essential to systematically evaluate key parameters influencing MR locomotion.

Hence, we further investigated the correlation between MR propulsion behavior and both the strength and pitch angle of the applied gradient magnetic field. As shown in Fig. 3H, the velocity of the MR increased with the rise in magnetic field gradient. The velocity variations of the MR under different pitch angles of the magnetic field, with a fixed field strength of 10 mT, are shown in Fig. 3I. The velocity exhibited a trend of first increasing and then decreasing as the pitch angle increased, peaking at a pitch angle of 10° with a maximum speed of 16 μm/s. This phenomenon may be attributed to the vertical gradient forces lifting the MRs, thereby reducing friction with the substrate. Additionally, based on $F_{\text{viscous}}=6\pi \eta rv\left(1+2r/h\right)$, as the height increased, the drag caused by interfacial effects diminished, further contributing to the increase in velocity [56], where *F*_viscous_ represents the viscous drag acting on the MR, *η* denotes the dynamic viscosity of the fluid, *r* is the radius of the MR, *v* represents the relative velocity of the MR with respect to the fluid, and *h* is the shortest distance between the MR and the wall. However, higher pitch angles resulted in reduced horizontal gradient forces, thereby decreasing propulsion efficiency.

### Combination therapy of PTX and BCG for bladder cancer

**Fig. 4.**
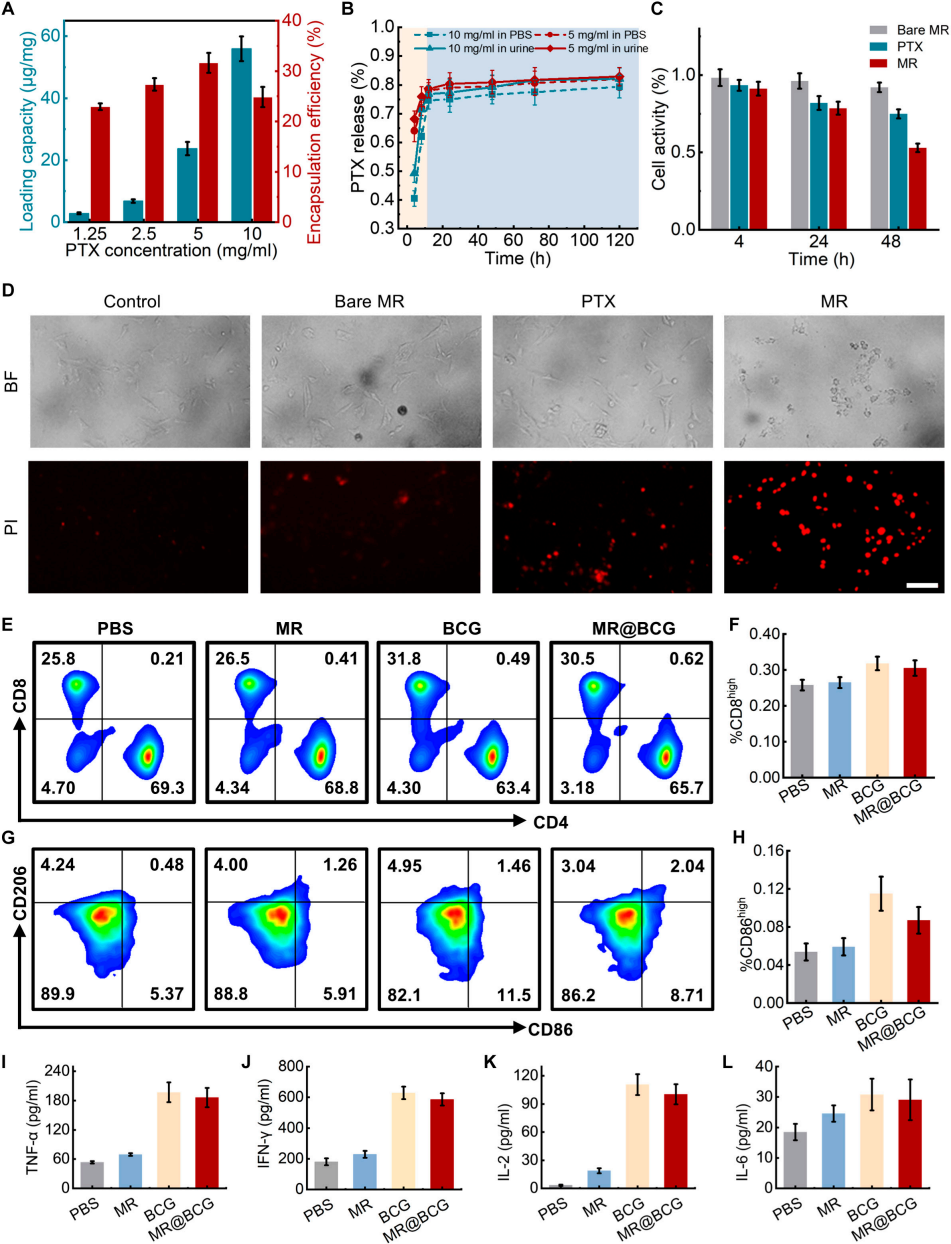
Drug release and tumor-killing effects of MR@BCG. (A) The loading capacity and encapsulation efficiency of PTX under different coating concentrations. (B) The release of free PTX from MRs over time in urine and PBS. (C) The tumor-killing effects of MR and PTX on bladder cancer MB49 cells. (D) Propidium iodide (PI) staining of MB49 cells treated with PBS, bare MRs, PTX, and MRs with PTX, respectively. Scale bar: 200 μm.(E to H) Expression of maturation markers of CD4^+^, CD8^+^, CD86^+^, and CD206^+^ after 7 d of incubation with MR@BCG or control samples. (I to L) Concentrations of tumor necrosis factor-α (TNF-α), interferon-γ (IFN-γ), interleukin-2 (IL-2), and interleukin-6 (IL-6) after incubation with MR@BCG or control samples for 48 h.

Monotherapy frequently resulted in the development of drug resistance in tumors, rendering it inadequate for bladder cancer treatment. Intravesical combination therapy using BCG and chemotherapeutic agents has emerged as a more effective approach for bladder cancer treatment. In this study, the PLGA layer of the MRs was loaded with the anticancer drug PTX, while BCG was adsorbed onto the surface of the CS layer via electrostatic interactions. Under an external magnetic field, the MRs exhibited directional movement, enabling the targeted delivery of both PTX and BCG to tumor sites for enhanced antitumor therapy. To quantify the drug loading and encapsulation efficiency of the MRs, various concentrations of PTX were encapsulated in the MRs. Testing PTX concentrations ranging from 1.25 to 10 mg/ml in the PLGA–PTX aqueous solution showed an increase in PTX dose per milligram of MR from 2.87 to 55.96 μg/mg (Fig. 4A). However, the encapsulation efficiency did not increase proportionally with increasing PTX concentration. At a PTX concentration of 10 mg/ml, the encapsulation efficiency decreased by approximately 10% compared to that at 5 mg/ml. Furthermore, the drug release characteristics of the MRs were investigated by placing drug-loaded MRs in PBS and urine and periodically sampling the supernatant for analysis. HPLC was used to measure the PTX concentration in the solution, constructing the drug release curve shown in Fig. 4B. The release rate was highest within the first 12 h, with approximately 75% of PTX released. Following this initial burst, the release rate gradually decreased over time. On the other hand, PTX exhibited a slightly higher release rate in urine than in PBS, likely reflecting the higher and variable osmolality of urine and the presence of urea/small solutes, which increase microsphere water uptake and polymer chain mobility (mild plasticization), thereby accelerating diffusion [57–59].

To identify a dose that maximizes antitumor efficacy while minimizing immunosuppression, we first characterized the PTX dose–response profiles in both immune effector cells and bladder tumor cells (Fig. S8). Within the identified therapeutic window, tumor inhibition exceeded 50% while immune cell viability remained above 90%. To evaluate the antitumor efficacy of the drug-loaded MRs, MB49 cells were treated with MRs loaded with concentrations of PTX. After 48 h of co-culture, MRs exhibited a cytotoxic effect on MB49 cells, which was stronger than that of free PTX (Fig. 4C). The bare MRs exhibited some inherent anticancer activity, attributable to hydrogen gas release. As shown in Fig. S9, the MRs demonstrated relatively mild cytotoxicity toward normal bladder epithelial cells. Additionally, PI staining showed that MR loaded with PTX exhibited potent cytotoxic effects on bladder tumor cells (Fig. 4D).

To assess the immunogenicity of MR@BCG, its effects on macrophages and T cells were evaluated in vitro using immune cells isolated from C57BL/6 mice. Immune cells were harvested from the mice and subsequently co-cultured with different samples. After 48 h of incubation, the cells were collected and analyzed by flow cytometry to evaluate the expression of maturation markers, as shown in Fig. 4E to H. The MR@BCG-treated group exhibited a noticeable increase in the expression of the CD8^+^ T cell marker and the M1 macrophage marker CD86^+^, indicating enhanced activation of cytotoxic T cells and proinflammatory macrophages. These results suggest that MR@BCG promotes antitumor immune responses by facilitating immune-cell-mediated tumor suppression. These levels were comparable to those induced by free BCG, indicating that the immunostimulatory potential of BCG was preserved after being loaded onto the MR. In contrast, MRs without BCG showed no marked differences relative to the control group. Furthermore, proinflammatory cytokine secretion was analyzed. Fig. 4I to L shows noticeable increases in TNF-α, IFN-γ, IL-2, and IL-6. In contrast, MRs without BCG maintained baseline levels of cytokines. Overall, MR@BCG effectively activated the innate immune response due to the inherent immunogenicity of BCG, facilitating a combination of immune- and drug-based therapy for tumor treatment.

### The in vitro navigation and fixation

**Fig. 5.**
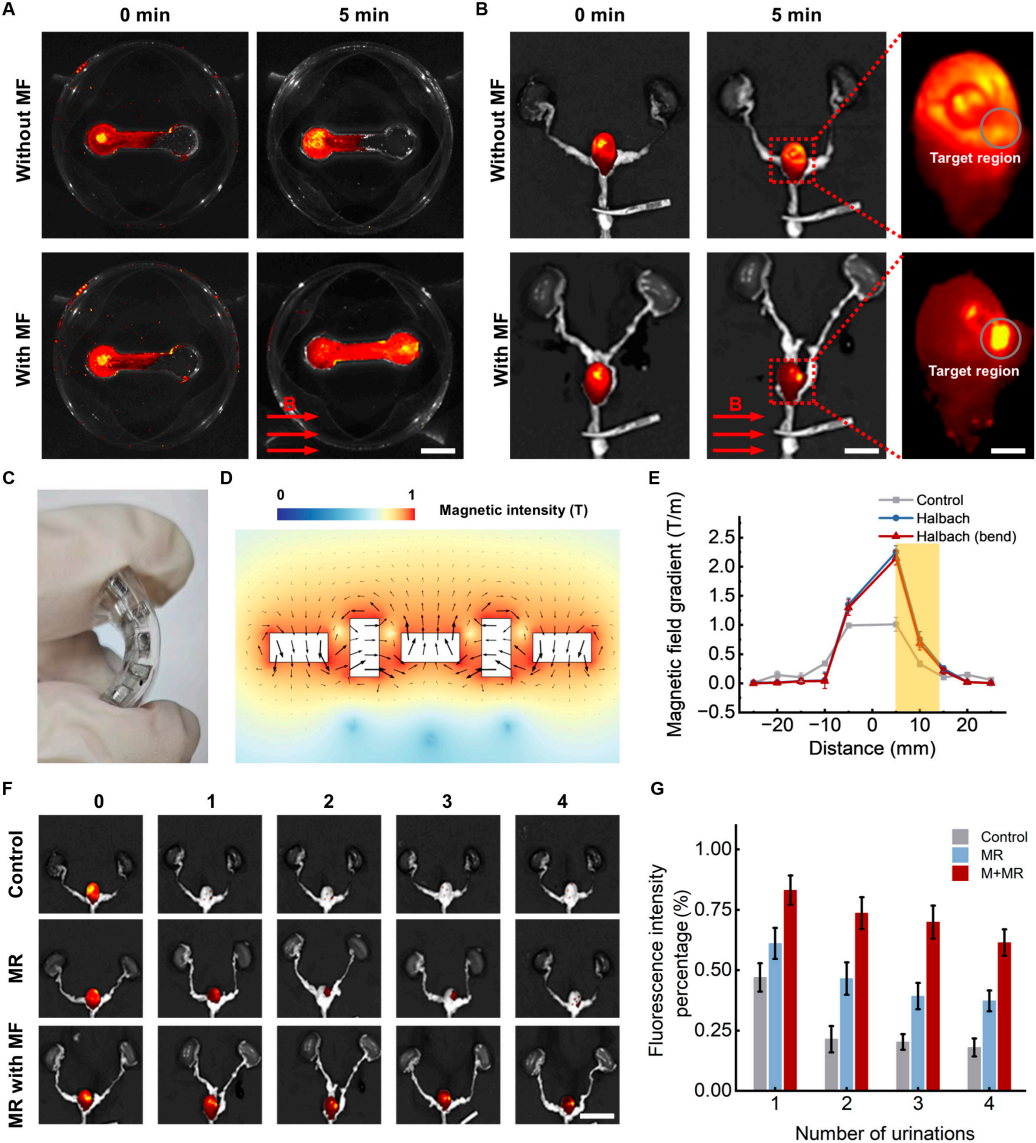
The in vitro navigation and fixation control of MRs driven by a magnetic field. (A) Fluorescence images of DiR-labeled MRs in the microchannel model under magnetic field control. Scale bar: 5 mm. (B) Ex vivo fluorescence images of DiR-labeled MRs in the urinary system dissected from mice without or with magnetic field control. Scale bars: 5 and 1 mm. (C) Wearable magnetic fixation device based on a Halbach array. (D) Magnetic field strength simulation of the Halbach array. (E) Relationship between the magnetic field strength of the Halbach array and distance from the array surface. (F) Fluorescence images of MRs retained in an ex vivo bladder with and without the magnetic fixation device. Scale bar: 1 cm. (G) Fixation rates of MRs in an ex vivo bladder without and with the magnetic fixation device. MF, magnetic field; M+MR, magnetic fixation device + magnetic microrobot.

A single MR was insufficient to address the therapeutic demands of bladder cancer. Therefore, we investigated the swarm behavior of MRs under magnetic field control. Initially, the targeted movement and fixation of MR swarms were assessed in microchannels and ex vivo bladder models. As shown in Fig. 5A, the microchannel consisted of 2 chambers, each with a radius of 3 mm, connected by a rectangular channel that was 10 mm long and 5 mm wide. DiR-labeled MRs were injected into the left chamber of the microchannel. In the absence of a magnetic field, the MRs gradually diffused over time but were unable to effectively move to the right chamber. After activating the horizontal gradient magnetic field, fluorescence imaging revealed that the MRs were gradually migrated to the right chamber within 5 min. This demonstrated the successful directional control and aggregation of MRs.

Furthermore, we evaluated the magnetic locomotion of MR swarms within the bladders of mice (Fig. 5B). DiR-labeled MRs were injected into the bladder via intravesical instillation through the urethra. The MRs gradually diffused and became uniformly distributed within the bladder without the magnetic field. However, upon the application of a magnetic field, a noticeable accumulation of MRs was observed in the target region, where high-intensity fluorescence signals were detected. The locomotion and migration of MR swarms in the microchannel and ex vivo bladder models were quantified, as shown in Fig. S10. Under magnetic field control, the fluorescence intensity in the targeted area accounted for 40% of the total, representing a 4-fold increase in targeting efficiency compared to random motion without a magnetic field. The magnetic moments of the MRs aligned with the magnetic field direction, enabling them to move toward the target under the propulsion of bubbles. By appropriately configuring the magnetic field parameters, the gradient magnetic force further accelerated the movement of the MRs.

The intravesically injected MRs could be easily cleared from the bladder under various physiological processes such as urination, which markedly shortened the residence time of the MRs. Therefore, after the successful targeted actuation of the MRs using the intelligent navigation system, a fixation device was necessary to immobilize MRs located at the targeted area. Unlike the MR navigation process, which required highly precise control of the magnetic field direction, the fixation of MRs required a strong local magnetic field gradient while maintaining portability and stability [60]. Therefore, as shown in Fig. 5C, a flexible wearable Halbach array was fabricated using rare-earth magnets and polydimethylsiloxane as the substrate for the fixation of MRs in the tumor region. The superposition of magnetic fields generated a strong magnetic field on the surface of the central permanent magnet. The magnetic field distribution in the surrounding area was calculated by COMSOL (Fig. 5D), revealing a marked increase in field strength in the direction of the central magnet due to the superposition effect. In contrast, the field strength decreased in the opposite direction due to cancellation. The wearable device underwent bending after being worn, and further simulations were conducted to calculate the magnetic field distribution generated by the Halbach array after bending. As shown in Fig. S11, there were no noticeable changes observed in the target area. Additionally, a magnetometer was used to measure the relationship between the magnetic field gradient and distance generated by the wearable fixation device before and after bending, as shown in Fig. 5E. An array consisting of 5 uniformly aligned permanent magnets was fabricated as a control group. Within the range of 5 to 15 mm (which corresponds to the typical distance between the mouse bladder and abdominal skin), the Halbach array generated a substantially stronger magnetic field distribution, reaching up to 23.52 mT, approximately twice that of the control group. In addition, the magnetic field gradient reached 2.24 T/m, which is more than twice that of the control group. This demonstrated its ability to achieve stronger and more stable MR fixation, greatly extending the drug retention time and enhancing drug utilization efficiency.

Furthermore, we evaluated the retention capability of MRs under magnetic field control during multiple simulated urination cycles (Fig. 5F and G). DiR-labeled MRs and PLGA particles were injected into an ex vivo bladder model through the urethra. The bladder wall was then mechanically compressed, simulating the forces associated with urination. In the control group, where PLGA particles were employed, the fluorescence intensity within the bladder decreased to 47% after the first urination cycle. In contrast, the MR group, coated with cationic CS, exhibited superior tissue adhesion properties, resulting in a retention efficiency of 61%. Further enhancement in retention was achieved under the influence of a magnetic field, where the retention efficiency increased to 83%. After 4 urination cycles, the control group showed a marked reduction in retention, with only 18% of the particles remaining in the bladder. In contrast, the MR group exhibited a higher retention of 37%. When subjected to a magnetic field, the retention rate further improved to 61%. These results demonstrated the excellent targeted movement and retention capabilities of MRs in the bladder. Overall, these findings underscore the potential of magnetically controlled MRs to provide reliable, noninvasive bladder treatments, enhancing drug delivery precision and extending therapeutic durations.

### In vivo targeted delivery and immune activation

**Fig. 6.**
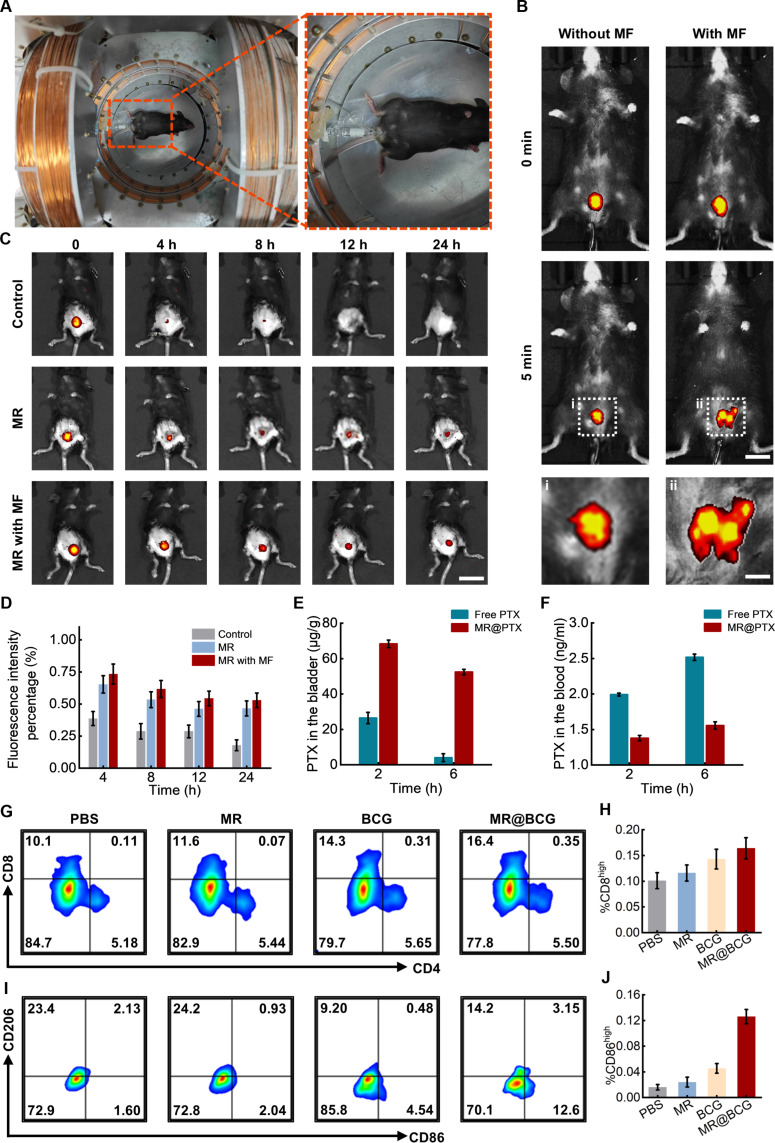
The in vivo navigation and fixation control of MRs driven by a magnetic field. (A) Magnetic field driving system for MRs. (B) In vivo imaging of post-intravesical DiR-labeled MR treated without or with magnetic field control. Scale bars: 1 cm and 4 mm. (C) Fluorescence images of an MR stationary in an in vivo bladder. Scale bar: 2 cm. (D) Fixation rates of MRs in an in vivo bladder without and with the magnet. (E) The amount of PTX absorbed into the bladder tissue. (F) Blood analysis of mice following intravesical instillation of PTX and MRs. (G to J) Proportions of immune cells in the bladder following stimulation with MR, BCG, and MR@BCG treatments.

To evaluate the targeting, retention, and drug release capabilities of MRs in vivo, we utilized a mouse bladder model and employed both in vivo fluorescence imaging and magnetic resonance imaging to monitor MR localization. As illustrated in Fig. 6A, MRs were injected into the bladder via the urethra using a catheter, and the mice were subsequently placed in the driving coil. In the absence of a magnetic field, MRs uniformly dispersed throughout the bladder, and the fluorescence intensity gradually decreased (Fig. 6B). Furthermore, magnetic resonance imaging confirmed the in vivo accumulation of the MRs in mice; as shown in Fig. S12, MR clusters produced prominent hypointense signals at the targeted site, verifying their aggregation at the intended location. Conversely, under the influence of a gradient magnetic field, MRs exhibited a progressive accumulation at the target site. Subsequently, a fixation device was used on the mice (Fig. S13), and MR retention was evaluated continuously over a 24-h period. Every 4 h, the fluorescence intensity was measured in the bladder to assess the retention of MRs, as shown in Fig. 6C and D. Similar to the in vitro experiments, the control group showed a noticeable reduction in fluorescence signal, which decreased by 38% after 4 h. However, the MR group exhibited an enhanced retention rate of 65%, attributed to its unique tissue adhesion properties. When subjected to a magnetic field, the retention rate further increased to 73%, and 53% of the MRs remained in the bladder after 24 h.

Further investigation was performed to evaluate the absorption of PTX in the bladder and serum, as shown in Fig. 6E and F. Two hours postinjection, the MR@PTX group exhibited a substantially higher PTX concentration in bladder tissues, attributed to its efficient retention capability. However, after 6 h, the free-PTX group showed a marked decline in tissue PTX levels, while the MR@PTX group demonstrated only a minimal decline. Additionally, after 6 h, the free-PTX group demonstrated an increase in PTX content in the serum, whereas the MR@PTX group exhibited a markedly lower concentration in the bloodstream. These findings suggested that MRs facilitate the retention of PTX in the bladder while minimizing systemic toxicity. This provided robust evidence for the potential of MR-based drug delivery systems in bladder cancer treatment, highlighting the efficacy of targeted, localized drug release in improving therapeutic outcomes.

Additionally, the bladders of treated mice were harvested, and a flow cytometer was employed to investigate the immune cell activation capability of microrobots in vivo (Fig. 6G to J). The results demonstrated that BCG effectively recruited T cells to the bladder and promoted the activation of CD8^+^ T cells. Furthermore, MR@BCG treatment further enhanced the infiltration and activation of CD8^+^ T cells compared with free BCG, indicating an improved local immune response. This enhancement can be attributed to the superior navigation and prolonged retention of the microrobots within the bladder. In parallel, the effect of microrobots on macrophage activation and polarization was assessed. Both BCG and MR@BCG induced macrophage polarization toward the M1 phenotype. Furthermore, the MR@BCG group exhibited a markedly higher proportion of CD86^+^ macrophages, reaching approximately twice that observed in the BCG group. These findings highlight the potential of microrobots in enhancing the efficacy of bladder cancer immunotherapy.

### MR@BCG inhibits tumor progression and prolongs survival in C57BL/6

**Fig. 7.**
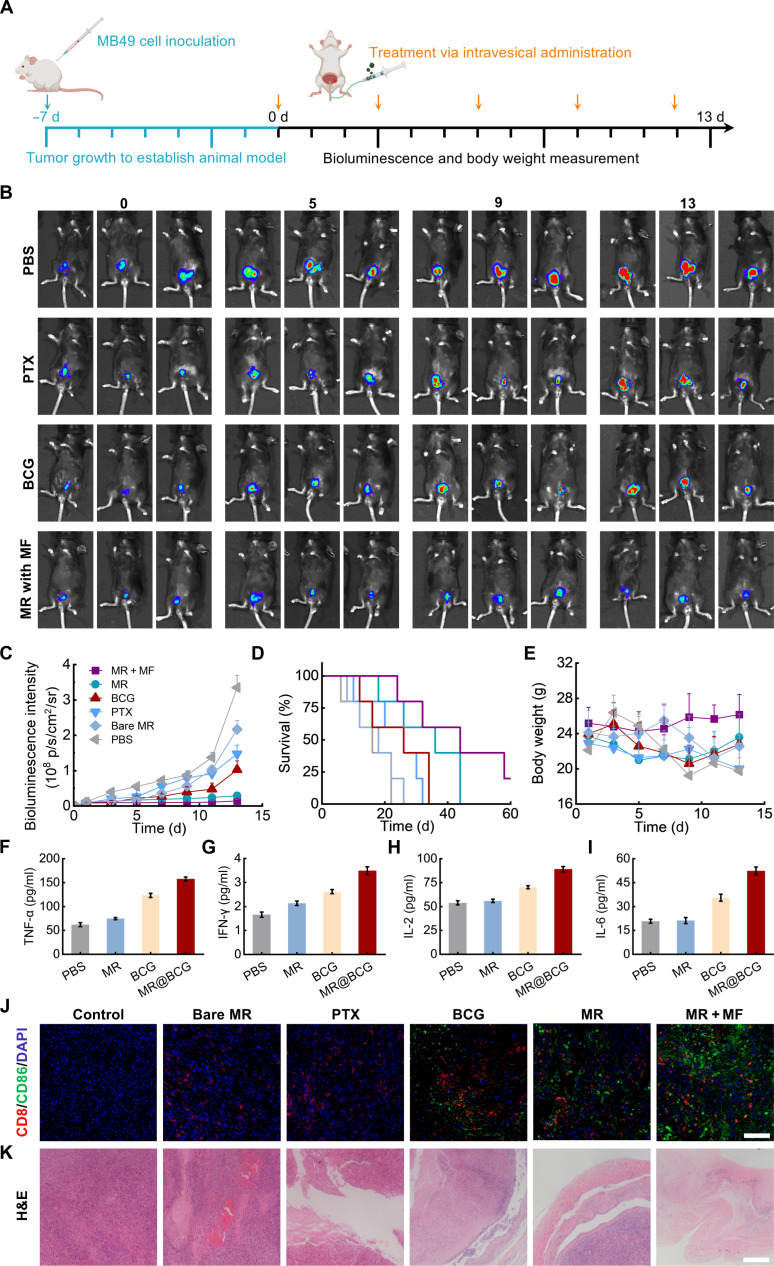
In vivo therapeutic efficacy of MR@BCG against bladder cancer. (A) Schematic of the study protocol in establishing orthotopic MB49 bladder tumors in C57BL/6 mice, followed by various intravesical administrations. (B) Representative in vivo bioluminescence images and (C) bioluminescence intensity of MB49-tumor-bearing mice under various intravesical administrations over the treatment course. (D) Survival rate and (E) body weight of mice under various intravesical administrations over the treatment course. (F to I) Concentrations of TNF-α, IFN-γ, IL-2, and IL-6 in bladder tissues after treatment with MR@BCG or control samples. (J) Immunofluorescence images showing CD8^+^ and CD86^+^ cells. Scale bar: 70 μm (K) Hematoxylin and eosin (H&E) staining images of bladder tissue. Scale bar: 125 μm. DAPI, 4′,6-diamidino-2-phenylindole.

Building on the demonstration of enhanced targeting and retention capabilities of MRs within the mouse bladder under magnetic field manipulation, their therapeutic efficacy against bladder cancer was further evaluated. The LUC-expressing MB49 cells were inoculated on the bladder wall, which were subsequently sutured and incubated for 1 week to establish the orthotopic bladder tumor model. Subsequently, the mice bearing orthotopic MB49 bladder tumors were randomly assigned into 8 groups, receiving intravesical instillation of PBS (negative control), bare MR, free PTX, free BCG, MR, MR@PTX (without BCG) + MF, MR@BCG (without PTX) + MF, and MR@BCG + MF. The mice in each group received intravesical instillation every 3 d for a total of 5 sessions (Fig. 7A).

Tumor growth was monitored using bioluminescence signals generated by LUC throughout the treatment process, as shown in Fig. 7B and Figs. S14 and S15. The bioluminescence intensity in the bare MR group was almost equivalent to that of the control group, indicating a lack of marked tumor growth suppression by the bare MR. However, mice treated with free PTX and BCG showed a decrease in bioluminescence intensity. Under matched dosing and magnetic parameters, both MR@PTX + MF and MR@BCG + MF outperformed their respective free-drug counterparts (PTX or BCG alone), which benefited of magnetic targeting and retention. This tumor-killing efficacy was further enhanced by intravesical injection of MR@BCG (PTX + BCG). These results highlighted the enhanced therapeutic potential of MR@BCG-based combined therapy compared to single-agent drug or immune treatments. In addition, the active transport and retention capabilities of the MRs not only enhanced the targeting efficiency but also prolonged the residence time of PTX and BCG, thereby amplifying their anticancer effects. The MR@BCG + MF group showed the lowest bioluminescence intensity, which was attributed to the ability of MRs to perform targeted motion under magnetic field guidance. Additionally, the use of fixation devices extended the retention time of MRs within the tumor region, further enhancing their anticancer effects. Specifically, the procedure was divided into 2 steps. In the targeting step of the MR, after intravesical instillation of MRs via the urethra, the magnetic field driving system guided them to the tumor site, and the driving system was then removed; in the subsequent fixation step, a medical strap secured the Halbach array to the mouse’s abdomen, providing a stable, strong local magnetic field.

Representative bioluminescent intensity curves of mice from various treatment groups depicted in Fig. 7C demonstrated that the MR@BCG + MF group displayed the lowest bioluminescence intensity on day 13. Additionally, the antitumor efficacy was further evidenced by the body weight changes and survival curve, as shown in Fig. 7D. Mice treated with free PTX and free BCG alone died within 35 d, whereas those in the MR@BCG treatment group survived up to 44 d. Moreover, the overall survival was substantially improved in the MR@BCG + MF group, with the maximum survival time exceeding 80 d.

The systemic toxicity of each treatment group was evaluated in mice. As shown in Fig. 7E, no noticeable body weight loss occurred during the study, and hematology/serum biochemistry indices (white blood cell count, red blood cell count, platelet count, globulin, total protein, and total bilirubin, Fig. S16) remained within normal ranges, showing no noticeable differences with controls. Collectively, these findings indicate good biocompatibility. Furthermore, mouse bladder tissues were harvested and cytokine levels were quantified, as shown in Fig. 7F to I. The PBS and bare MR groups showed no noticeable differences, with TNF-α, IFN-γ, IL-2, and IL-6 remaining at baseline. Free BCG induced a clear increase in all 4 cytokines. Strikingly, MR@BCG produced the highest upregulation, indicating that the MRs’ targeted delivery and prolonged retention of BCG further amplified innate immune activation. Immunostaining for CD86^+^ and CD8^+^ cells was performed to investigate immune cell enrichment within the bladder. As shown in Fig. 7J, BCG substantially activated immune cells. Both the MR@BCG and MR@BCG + MF groups exhibited greater immune cell activation, attributable to the MRs’ active navigation and prolonged retention. Tumor growth in the bladders was further examined using Ki-67 and hematoxylin and eosin staining, and as illustrated in Fig. 7K and Fig. S17, mice treated with MR@BCG + MF exhibited a noticeable reduction in bladder tumor size compared with those receiving other treatments.

## Discussion

Despite remarkable progress in the development of MR systems, several key challenges have continued to hinder their translation into practical biomedical applications. First, achieving efficient and precise propulsion under physiological conditions has remained a noticeable hurdle [51]. While chemically driven MRs with biocompatible fuels (e.g., water and urea) have exhibited effective motility [22,26], their movement has typically been restricted by gradient-directed chemotaxis, lacking controllable navigation. In contrast, magnetically actuated MRs have demonstrated impressive control capabilities in vivo [32,48]; however, overcoming the high viscosity and complex biological environment has often required strong magnetic fields, which have led to considerable heat generation during prolonged operation. Moreover, increasing the magnetic responsiveness has necessitated a higher magnetic material content, posing fabrication and scalability challenges. Second, maintaining stable retention at the target site in the presence of physiological clearance mechanisms, such as urination, has been particularly challenging for urinary tract applications. Although existing systems have improved tissue adhesion through surface modifications or specialized structural designs [6,56], the development of more universally applicable and robust retention strategies has remained imperative. Third, designing therapeutic strategies that have enhanced antitumor efficacy while mitigating drug resistance and interpatient variability has remained an urgent clinical need [46].

To address these limitations, we developed a hybrid-propulsion MR delivery system that combined chemically induced hydrogen bubble propulsion with magnetic field navigation to enable efficient and precise 3-dimensional locomotion. In addition, we designed a wearable magnetic fixation device based on the Halbach array to reinforce MR retention at the tumor site, thereby facilitating sustained release for synergistic chemotherapy and immunotherapy.

Using a layer-by-layer assembly approach, we successfully fabricated MRs co-loaded with PTX, BCG, and Fe_3_O_4_ nanoparticles. For precise in vitro navigation, we designed a sliding mode controller integrated with an extended state observer, which notably improved directional control under dynamic magnetic fields. Furthermore, the wearable Halbach array markedly enhanced local magnetic field strength, extended MR retention time within dynamic physiological environments such as the bladder, and substantially increased local drug release efficiency. Our system demonstrated the synergistic therapeutic effects of PTX-induced cytotoxicity and BCG-mediated immune stimulation, substantially enhancing antitumor efficacy and reducing drug resistance in a murine bladder cancer model. Additionally, the use of magnesium and PLGA conferred essential biodegradability, ensuring both in vivo safety and long-term biocompatibility—attributes that were confirmed through in vivo testing.

Looking ahead, the successful clinical translation of the MR@BCG MR system will require scalable manufacturing techniques and consistent batch-to-batch quality control. Moreover, real-time in vivo tracking and imaging have remained critical technological bottlenecks that must be addressed. In future work, we will explore higher-frame-rate modalities (such as ultrasound-based imaging) to overcome these limitations and enable real-time in vivo control. Meanwhile, sequential dosing through rational microarchitectural design represents a key strategy to mitigate PTX-mediated damage to newly recruited immune cells and improve overall tumor control. Future research should prioritize long-term safety evaluations and validation in large-animal models that more closely approximate human physiology. Overall, this hybrid-propulsion MR platform has provided a promising and versatile approach for targeted drug delivery and combination cancer therapy, offering a robust foundation for the development of clinically translatable MR systems for bladder cancer treatment.

## Conclusion

In this study, we developed an MR-based targeted delivery system to address the limitations of bladder cancer therapies. Using a layer-by-layer assembly strategy, MRs were successfully fabricated to load PTX, BCG, and magnetic particles, achieving precise navigation, stable retention, and synergistic therapeutic effects. The MRs exhibited self-propulsion by reacting with water to generate hydrogen bubbles and were accurately guided by external magnetic fields, enabling efficient delivery to tumor sites. Upon reaching the target location, the combined chemotherapeutic and immunostimulatory effects inhibited tumor progression and substantially prolonged the survival of mice with orthotopic bladder cancer. The incorporation of a flexible wearable fixation device based on a Halbach array generated a stable, enhanced magnetic field, which fixated the MRs at the tumor region, extending their retention time and further improving therapeutic efficacy. The proposed system demonstrated noticeable advantages in precision, retention, and therapeutic performance compared to traditional methods, highlighting its potential for clinical translation in intravesical cancer therapy. This work not only provided a promising platform for bladder cancer treatment but also contributed to the broader application of MR-based systems in targeted drug delivery and combination therapy.

## Data Availability

The data that support the findings of this study are available from the corresponding authors upon reasonable request.

## Supplementary Materials

Supplementary 1 Texts S1 and S2 Figs. S1 to S17 Movies S1 to S4
