# Supplementary material for: Hybrid-Driven Bacillus Calmette–Guérin Carrier for Targeted Immuno-Chemo Combo Therapy in Bladder Cancer
Source: Cyborg Bionic Syst. 2026 Apr 28;7:0492. doi: 10.34133/cbsystems.0492 (PMC13121890; doi:10.34133/cbsystems.0492)
Supplement: Supplementary 1 — Texts S1 and S2 Figs. S1 to S17 Movies S1 to S4 [file cbsystems.0492.f1.zip › Supporting Information.docx]

Supporting Information

**Hybrid-driven BCG Carrier for Targeted Immuno-Chemo Combo Therapy in Bladder Cancer**

*Zhanxiang Zhang, Lin Wang, Zhongcheng An, Yuhang Jiang, Jiawen Niu, Laishou Yang, Qianqian Wang*, Xinjian Fan*, Tianlong Li**

**This PDF file includes:**

Supplementary Text S1 Composition of the microrobot control system.

Supplementary Text S2. ESO-based control strategy for disturbance estimation and precise motion regulation of microrobots.

Supplementary Fig. S1 Fluorescence images of MR@Dox.

Supplementary Fig. S2. Schematic autonomous control of MR.

Supplementary Fig. S3. Photograph of the experimental setup for the microrobot control system.

Supplementary Fig. S4. Variation of microrobot diffusion distance over time.

Supplementary Fig. S5. Comparison between predicted trajectory and actual path for autonomous navigation.

Supplementary Fig. S6. Angle error analysis during autonomous navigation.

Supplementary Fig. S7. Microrobot locomotion speed under different pH conditions.

Supplementary Fig. S8. Dose-dependent effects of paclitaxel on immune effector and bladder tumor cell viability.

Supplementary Fig. S9. Effects of microrobots and paclitaxel on urothelial cells viability.

Supplementary Fig. S10. Proportion of fluorescence intensity in the targeted region.

Supplementary Fig. S11. Simulation of magnetic field surrounding the curved Halbach array.

Supplementary Fig. S12. MRI of microrobots with and without magnetic actuation.

Supplementary Fig. S13. Photograph of a mouse wearing the Halbach array.

Supplementary Fig. S14. In vivo bioluminescence imaging of MB49 tumor-bearing mice at days 3, 7, and 11 under different treatment conditions: PBS (control), PTX, BCG, and microrobot (MR)-mediated therapy.

Supplementary Fig. S15. Representativein vivo bioluminescence images of MB49 tumor-bearing mice under intravesical infusion of bare MR and MR treatment conditions.

Supplementary Fig.S16 Hematology (WBC, RBC, PLT) and serum biochemistry (GLOB, TP, T-Bil) after treatment.

Supplementary Fig. S17. Ki67 images of bladder tissue. Scale bar :125 μm.

**Other Supplementary Materials for this manuscript include the following:**

Movie S1. Self-correction of motion deviation in microrobot navigation.

Movie S2. Controllable retention of microrobot under rotating magnetic field actuation.

Movie S3. Obstacle-crossing of a microrobot driven by gradient magnetic fields with adjustable pitch angles.

Movie S4. Shell-only microrobot locomotion under magnetic field after complete Mg-core reaction

**Text S1. Composition of the microrobot control system.**

The system used a microscope-coupled CCD camera to capture the top view of the workspace, providing the real-time feedback. The video stream was processed by a tracker comprising object tracking and state estimation algorithms, which extracted the microrobot's position and velocity states and relayed them to a PC for further processing. A controller with decoupled direction and speed was designed, incorporating an extended state observer (ESO) to estimate the bubble propulsion force as well as the varying rotational inertia and mass of the microrobot. To enhance the system's robustness, a sliding mode controller (SMC) was employed to effectively compensate for these variations and mitigate external disturbances. The controller dynamically adjusted its parameters based on the current motion state, generating driving signals to correct direction and speed errors. An I/O card was integrated to convert the digital control signals from control PC into analog voltage signals, which were amplified by a power amplifier and supplied to coils. Finally, the driving coils generated magnetic fields for the precise navigation of microrobot.

**Text S2. ESO-based control strategy for disturbance estimation and precise motion regulation of microrobots.**

First, we construct the dynamic model of the system as follows:

Where:

: propulsion force generated by bubble ejection.

: the current orientation of the microrobot.

: the angle between the jet propulsion force and the microrobot's current orientation.

and : X- and Y-components of the force induced by the magnetic field gradient.

: the offset distance of mass.

: torque induced by the external magnetic field.

: translational damping coefficient.

: rotational damping coefficient.

, , and could be calculated as follows:

Where *Gxx, Gxy, Gyx, Gyy*: Components of the magnetic field gradient matrix.

*mr*: magnetic moment of the robot.

*B*: magnitude of the external magnetic field.

*θB​*: direction of the external magnetic field.

The parameters *Fprop*, *δ*, *δr* were considered as disturbances, and an Extended State Observer (ESO) was constructed to estimate these parameters by treating them as part of the system's extended states.

**ESO for X-direction:**

**ESO for Y-direction:**

**ESO for Attitude Dynamics:**

Therefore, *Fprop*, *δ*, *δr* could be calculated using the following equation:1,2

Based on this, sliding mode controllers (SMCs) were separately designed for the microrobot's attitude and translational motion.

**Attitude control methods:**

The attitude error is defined as:

The sliding surface is constructed as:3,4

**Motion control methods**

The position and velocity errors along the x and y directions were defined separately as follows:

Then, the sliding surface was defined by:

The desired compensation forces in the x- and y-directions are chosen to be:

The magnetic field gradient was calculated as follows:

**Supplementary Fig.**

**
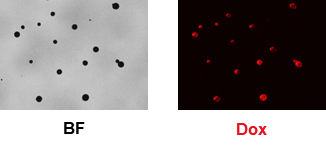
**

**Fig. S1. Fluorescence images of MR@Dox.**


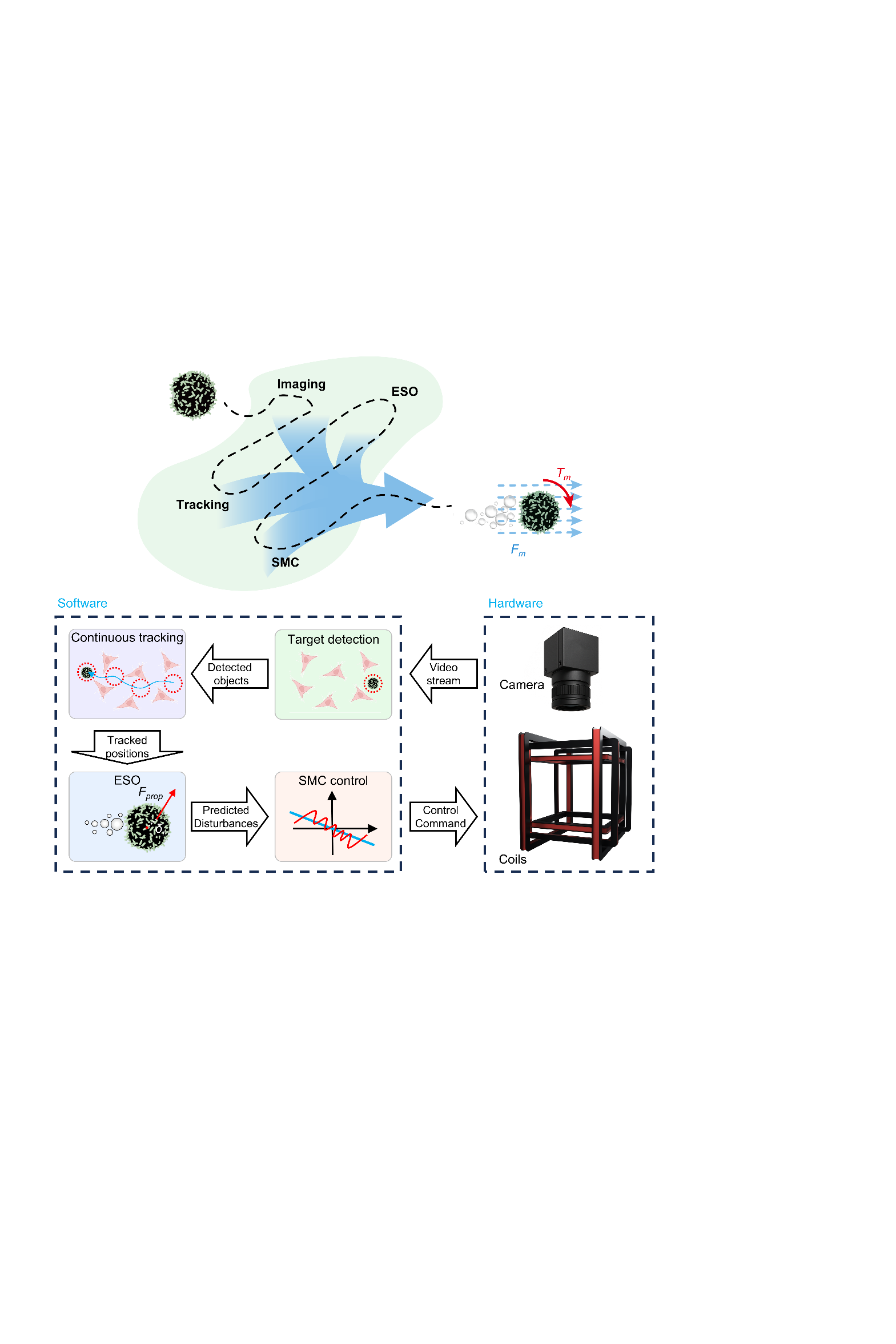


**Fig. S2. Schematic autonomous control of MR.**

**
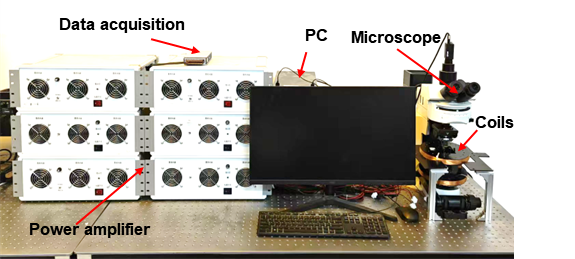
**

**Fig. S3. Photograph of the experimental setup for the microrobot control system.**

**
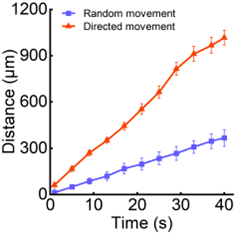
**

**Fig. S4. Variation of microrobot diffusion distance over time.**

**
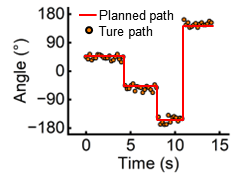
**

**Fig. S5. Comparison between predicted trajectory and actual path for autonomous navigation.**

**
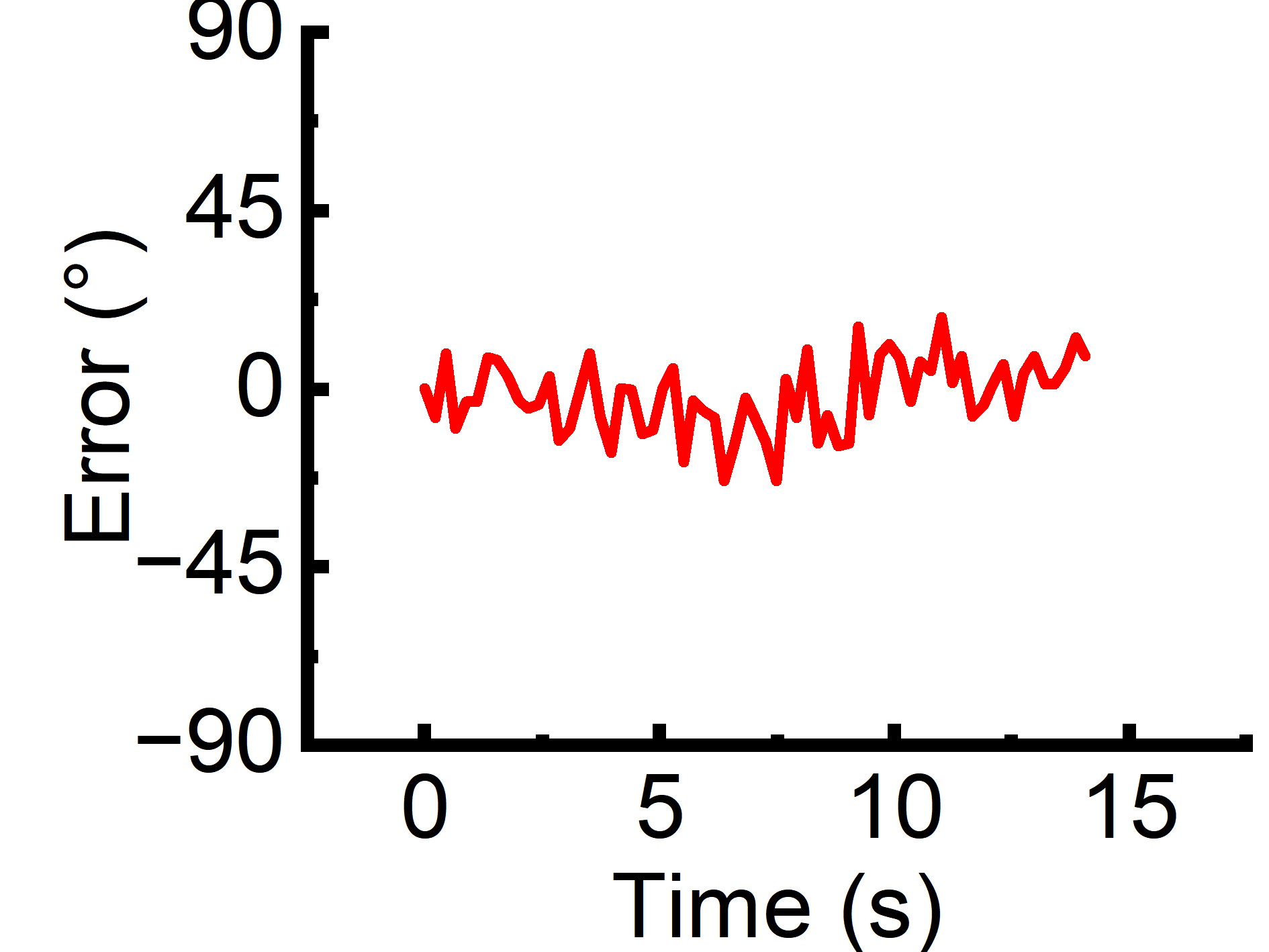
**

**Fig. S6. Angle error analysis during autonomous navigation.**

**
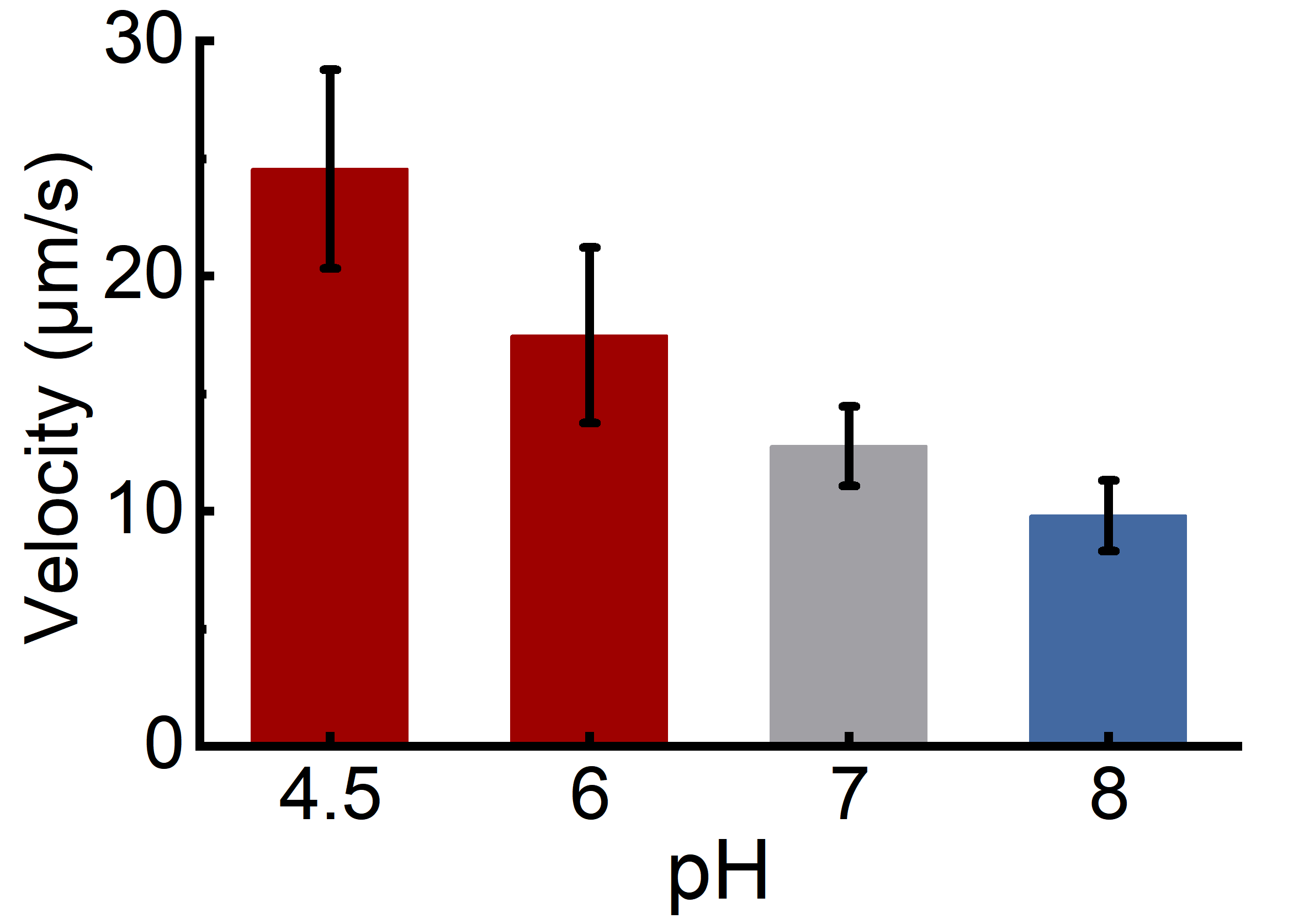
**

**Fig. S7. Microrobot locomotion speed under different pH conditions.**

**
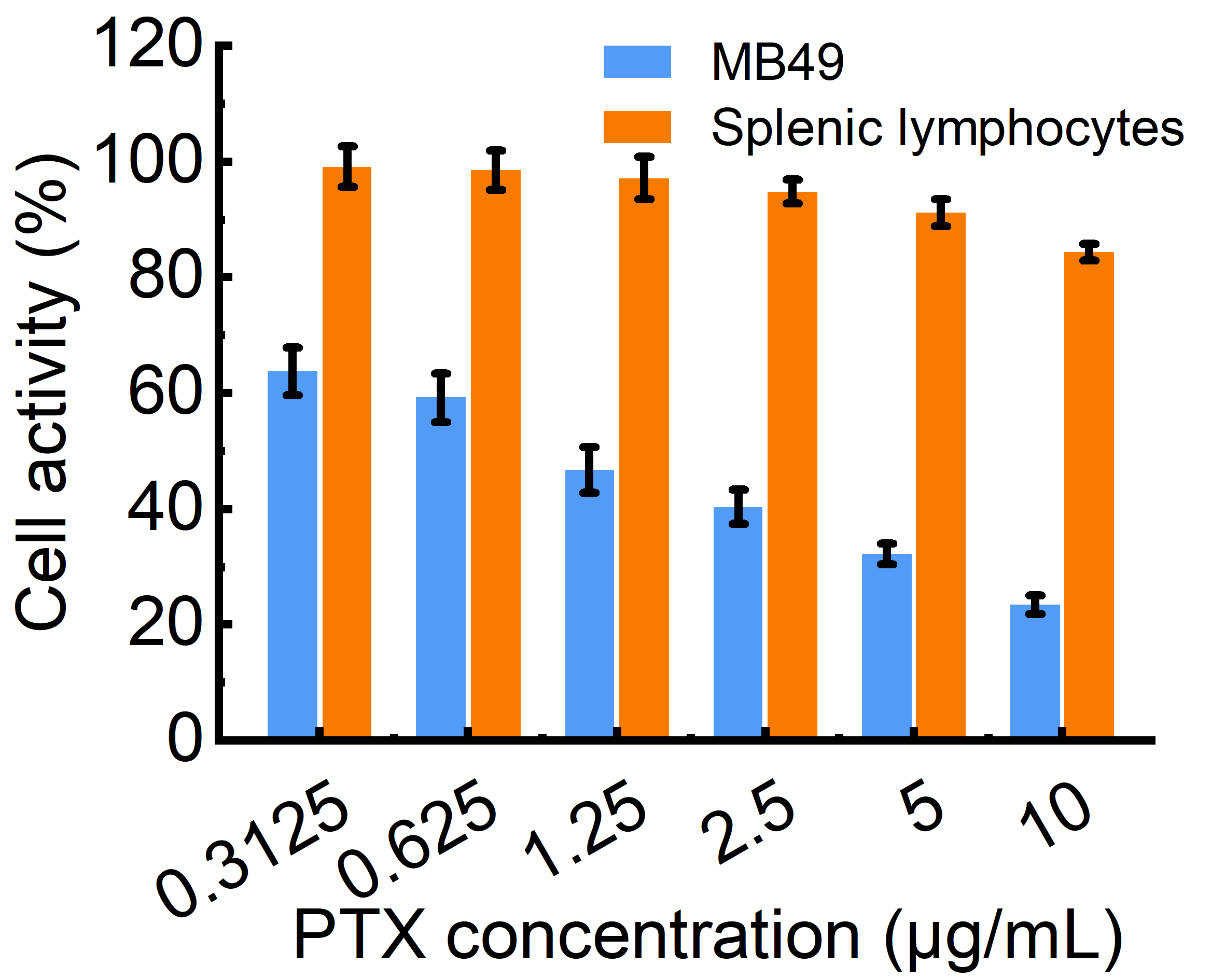
**

**Fig. S8. Dose-dependent effects of paclitaxel on immune effector and bladder tumor cell viability.**

**
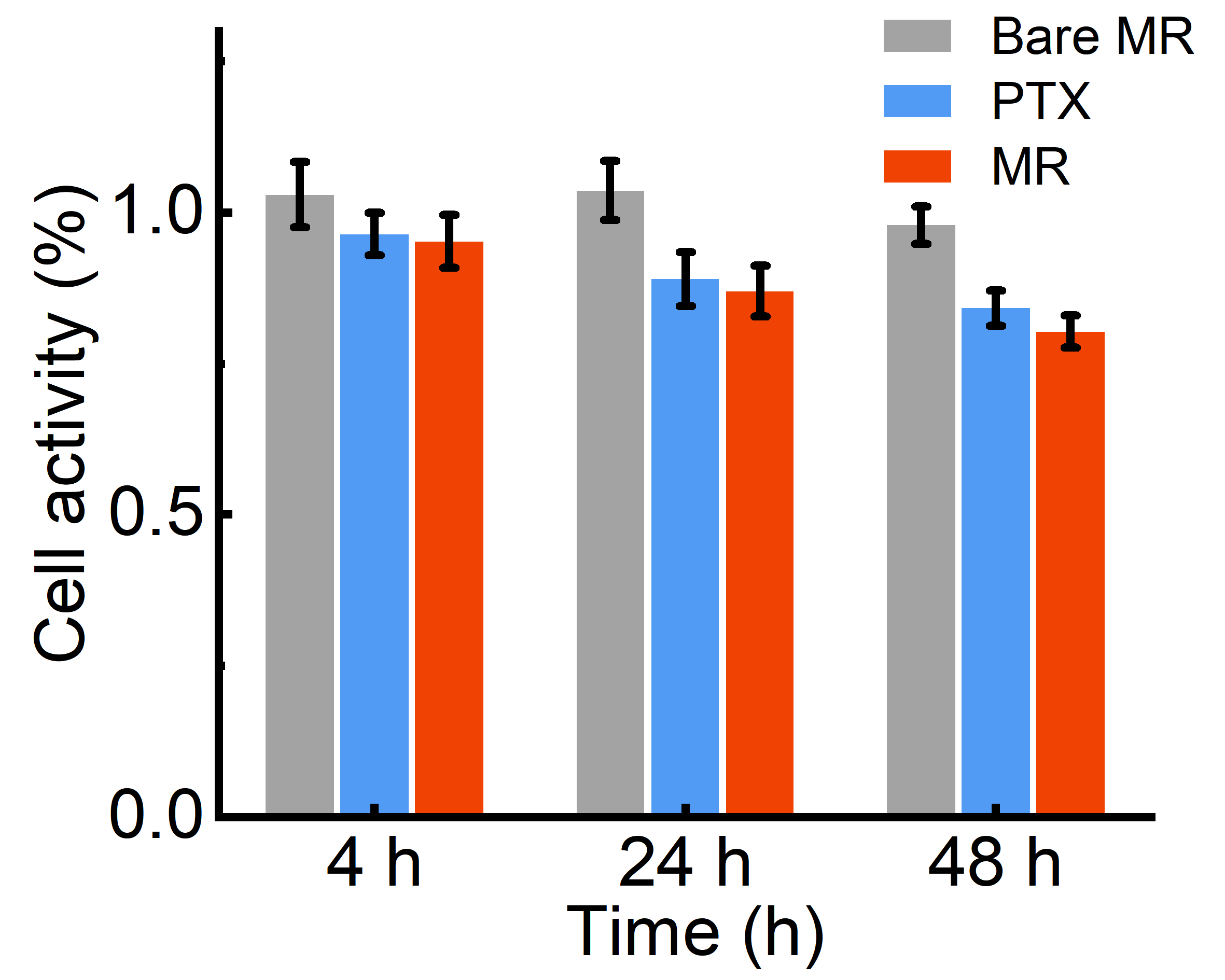
**

**Fig. S9. Effects of microrobots and paclitaxel on urothelial cells viability.**

**
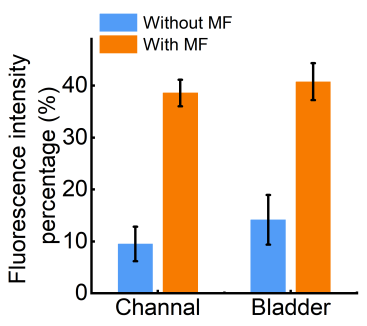
**

**Fig. S10. Proportion of fluorescence intensity in the targeted region.**

**
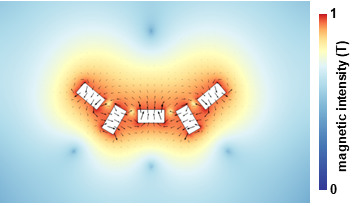
**

**Fig. S11. Simulation of magnetic field surrounding the curved Halbach array.**

**
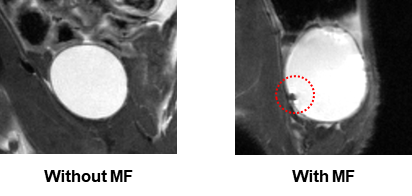
**

**Fig. S12. MRI of microrobots with and without magnetic actuation.**

**
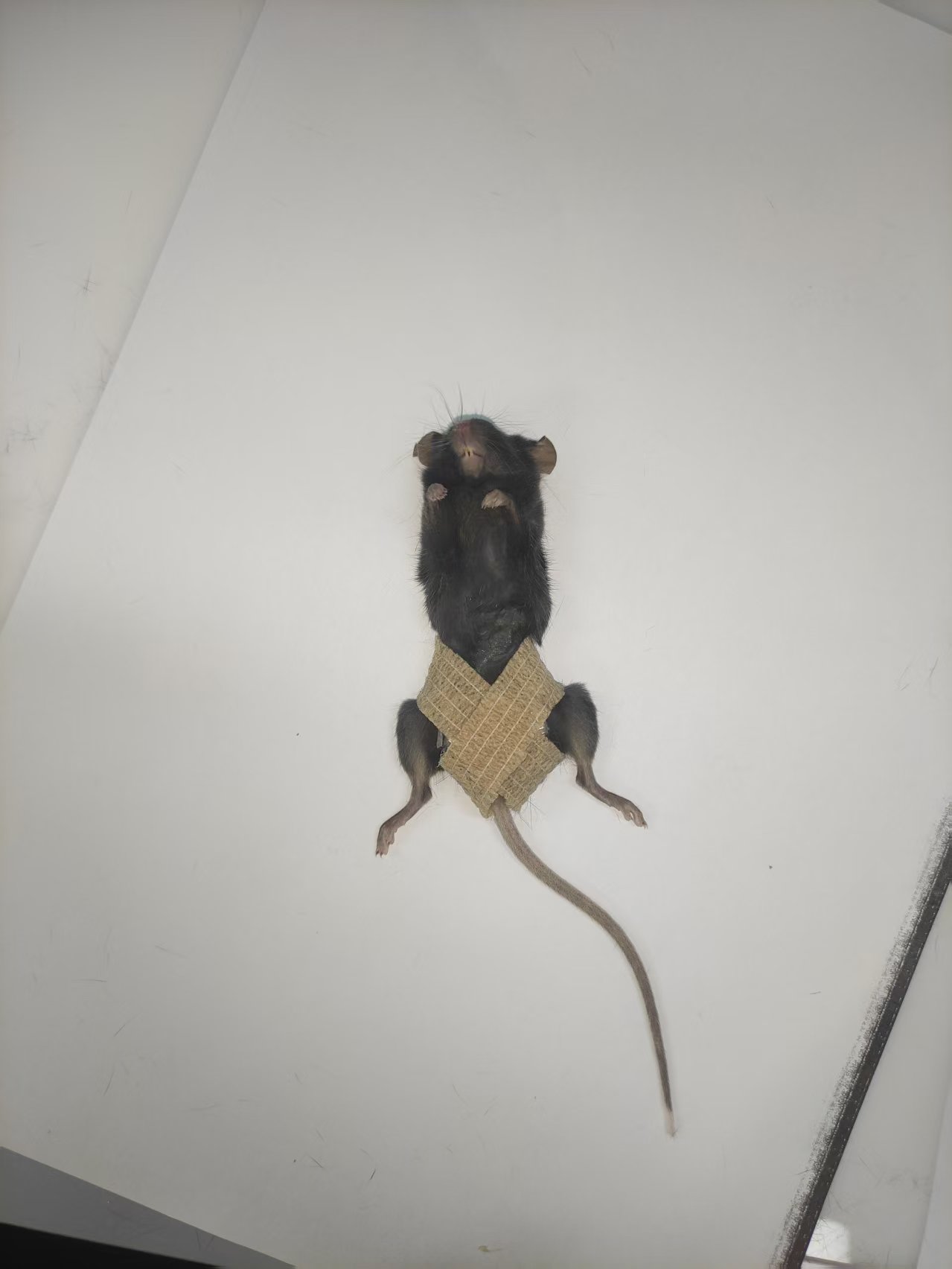
**

**Fig. S13. Photograph of a mouse wearing the Halbach array.**

**
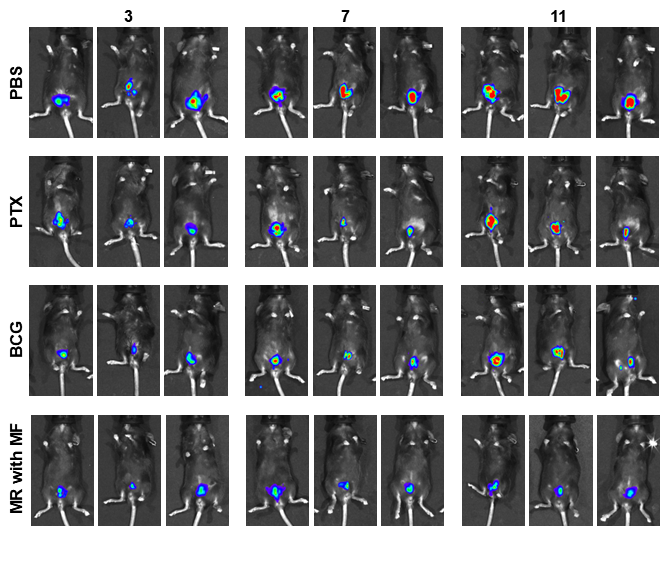
**

**Fig. S14. In vivo bioluminescence imaging of MB49 tumor-bearing mice at days 3, 7, and 11 under different treatment conditions: PBS (control), PTX, BCG, and microrobot (MR)-mediated therapy.**

**
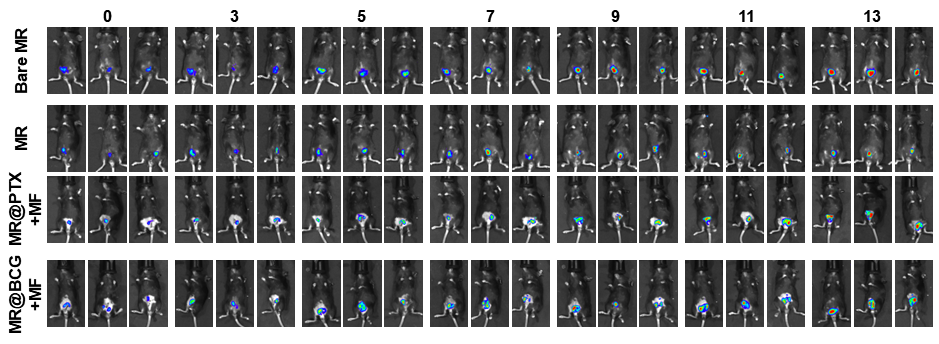
**

**Fig. S15. Representative in vivo bioluminescence images of MB49 tumor-bearing mice under intravesical infusion of bare MR, MR, MR@PTX+MF and MR@BCG+MF treatment conditions.**


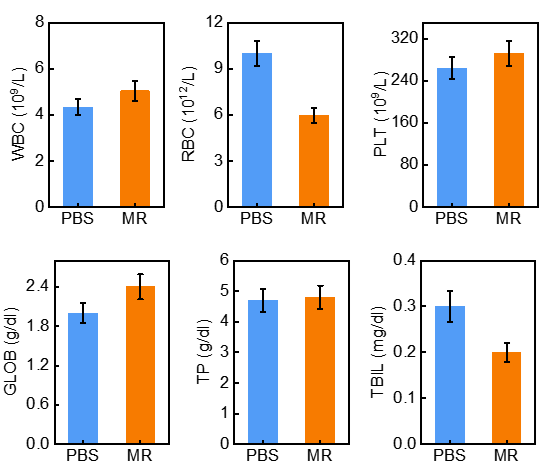


**Fig.S16 Hematology (WBC, RBC, PLT) and serum biochemistry (GLOB, TP, T-Bil) after treatment.**

**
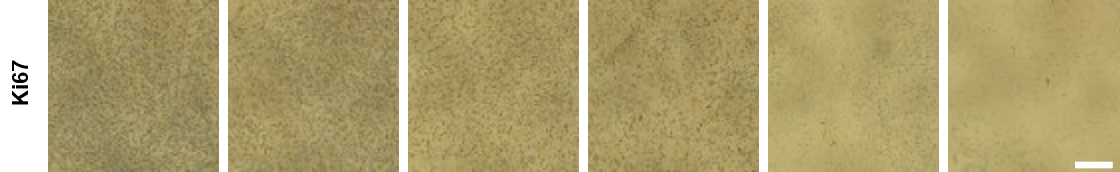
**

**Fig. S17. Ki67 images of bladder tissue. Scale bar :125 μm.**

**References**

1 Lu, J. *et al.* Robust control strategy of gradient magnetic drive for microrobots based on extended state observer. *Cyborg and Bionic Systems* (2022). <https://doi.org/10.34133/2022/9835014>

2 Liu, Y. *et al.* Autonomous navigation of magnetic microrobots with improved planning and control in complex environments. *IEEE Transactions on Automation Science and Engineering* (2024). <https://doi.org/10.1109/TASE.2024.3379364>

3 Jia, Y., Zheng, L., Dong, D., Wang, Y. & Sun, D. Robust navigation control of a microrobot with hysteresis compensation. *IEEE Transactions on Automation Science and Engineering* **19**, 3083-3092 (2021). <https://doi.org/10.1109/TASE.2021.3106022>

4 Mousavi, A., Ahmed, A., Khaksar, H., Choi, H. & Hoshiar, A. K. An input Saturation-Tolerant position control method for magnetic microrobots using adaptive fuzzy sliding-mode eethod. *IEEE Transactions on Automation Science and Engineering* (2024). <https://doi.org/10.1109/TASE.2024.3400602>
